# Mechanism of activation of an ancestral TEC kinase by PIP_3_

**DOI:** 10.1038/s44511-026-00001-z

**Published:** 2026-10-01

**Authors:** Eva Krötenheerdt, Lucas Piëch, Freia von Raußendorf, Ronja Reinhardt, Ranjana Nataraj, Nicholas Wedige, Hunter G. Nyvall, Emma E. Walsh, John E. Burke, Thomas A. Leonard

**Affiliations:** 1 Max Perutz Labs, Medical University of Vienna, Vienna, Austria; 2 Vienna BioCenter PhD Program, A Doctoral School of the University of Vienna and the Medical University of Vienna, Vienna, Austria; 3 BioOptics Light Microscopy Facility, Max Perutz Labs, Vienna, Austria; 4 Department of Biochemistry and Microbiology, University of Victoria, Victoria, BC Canada; 5 Department of Biochemistry and Molecular Biology, University of British Columbia, Vancouver, BC Canada; 6 University of Victoria Genome BC Proteomics Centre, Victoria, BC Canada

**Keywords:** Cell biology, Molecular biology

## Abstract

The TEC kinases are a family of five paralogous mammalian genes that play crucial roles in cell growth, proliferation, and differentiation, particularly in immune cells. The recruitment and activation of the TEC kinases depend on the generation of the lipid second messenger, PIP_3_, in the plasma membrane. However, the mechanisms by which PIP_3_ activates the TEC kinases are not well understood. By studying an ancestral TEC kinase from the choanoflagellate *Monosiga brevicollis*, we have elucidated an evolutionarily conserved mechanism for the activation of the TEC kinases by PIP_3_. We demonstrate that PIP_3_, but not PIP_2_, relieves autoinhibition by displacing the PH domain of MbTEC from a conserved intramolecular interaction with its kinase domain. We also show that a conserved polyproline motif within MbTEC promotes its activation in a kinase-intrinsic mechanism. Finally, we show that the PH domain is sufficient to restore autoinhibition in a constitutively active mutant of MbTEC. Our findings reveal that PIP_3_ is necessary but not sufficient for MbTEC activation.

## Introduction

The thymic epithelial cell (TEC) kinases are a subfamily of the Src family of non-receptor tyrosine kinases (SFK), best known for their roles in the proliferation and differentiation of immune cells^1^. Mutations in Bruton’s tyrosine kinase (BTK), one of the five mammalian TEC kinase paralogs, are associated with X-linked agammaglobulinemia (XLA), a disease characterized by a failure of pre-B cells to mature into terminally differentiated, antibody-producing B cells, and a consequent lack of circulating antibodies^2^. Mutations in the same gene lead to X-linked immunodeficiency in mice^3^. Conversely, inhibition of BTK is used in the clinic to treat a range of B cell malignancies, including chronic lymphocytic leukemia, mantle cell lymphoma, and Waldenström’s macroglobulinemia^4^.

The TEC kinases are distinguished by an N-terminal pleckstrin homology (PH) domain, which is appended to an ancient Src homology 3 (SH3)–Src homology 2 (SH2)-kinase (32K) module shared by all SFKs^5^. BTK is autoregulated by its SH3 and SH2 domains, which pack against the kinase domain to maintain it in an inactive conformation^6,7^, as also observed for SRC, hematopoietic cell kinase (HCK), and Abelson murine leukemia viral oncogene homolog 1 (ABL)^8–10^. The SH2 and SH3 domains bind to phosphorylated tyrosine marks and polyproline motifs in activated receptors at the plasma membrane, respectively. This leads to the specific recruitment of BTK to membrane proximal sites, which is a prerequisite for its activation and subsequent downstream substrate phosphorylation. Early reports suggested that the activation loops of BTK and inducible T cell kinase (ITK) are *trans*-phosphorylated by other SFKs^11–13^, while more recent reports have attributed activation loop phosphorylation to autocatalysis^6,7,14^.

Unlike other SFKs, which are localized to the plasma membrane via myristoylation of their N-termini^15^, the localization of the TEC kinases depends on the lipid second messenger phosphatidylinositol-3,4,5-trisphosphate (PIP_3_). PIP_3_ produced by class I phosphatidylinositol 3-kinases (PI3K) immediately downstream of the B cell receptor (BCR) or T cell receptor (TCR) is required for re-localization of BTK and ITK, respectively, from the cytosol to the plasma membrane. Various functions have been ascribed to the PH domains of BTK and ITK, from targeting to the plasma membrane^16–18^ to PIP_3_-mediated dimerization^6,14,19^ and autoinhibition of kinase activity^6,7,20–22^. While membrane targeting and co-localization of ITK with the TCR can be substituted with the membrane-targeting myristoylation sequence of LCK (lymphocyte-specific protein tyrosine kinase), the PH domain is indispensable for kinase activation^23^. Although full-length BTK has recently been crystallized, the PH domain was not resolved in the electron density map^7^, while studies of BTK in solution and by cryo-electron microscopy have concluded that the protein adopts a spectrum of conformations with varying degrees of compaction^7,24,25^. Together with the available experimental structures, mapping of the conformation of the PH domain in BTK and ITK by hydrogen–deuterium exchange mass spectrometry (HDX-MS) and nuclear magnetic resonance (NMR) spectroscopy has led to proposals of an ensemble of conformational states in solution^7,20,21,26^. The role, therefore, of the PH domain in regulating the activity of the TEC kinases is still incompletely understood.

The TEC kinases first emerged in unicellular choanoflagellates, before gene duplication, prior to the evolution of vertebrates, gave rise to the five mammalian paralogs^27^. Tissue-specific expression patterns, particularly in immune cells, have since given rise to the diversification of the signaling pathways regulated by individual paralogs^28^. To elucidate the mechanism by which the TEC kinases are regulated by PIP_3_, we studied an ancestral TEC kinase from the choanoflagellate *Monosiga brevicollis*, which we hereinafter refer to as MbTEC. We show that MbTEC adopts a compact conformation in solution and that the PH domain both stabilizes this conformation and inhibits its kinase activity. We provide evidence that the PIP_3_-binding pocket of the PH domain is sequestered in a specific autoinhibitory conformation centered on the kinase domain of MbTEC and that mutation of either side of the PH–kinase interface activates MbTEC independently of PIP_3_. Finally, we demonstrate that a conserved polyproline motif in MbTEC plays a crucial role in its allosteric, PIP_3_-mediated activation. Our findings reveal that PIP_3_ is necessary but not sufficient for MbTEC activation, thereby confining TEC activity to the membrane. TEC kinases are, therefore, coincident detectors of PIP_3_, phospho-tyrosine, and polyproline motifs, all of which must be engaged for the recruitment and activation of TEC with high fidelity.

## Results

### *Monosiga brevicollis* TEC adopts a conserved autoinhibited conformation

**Fig. 1:**
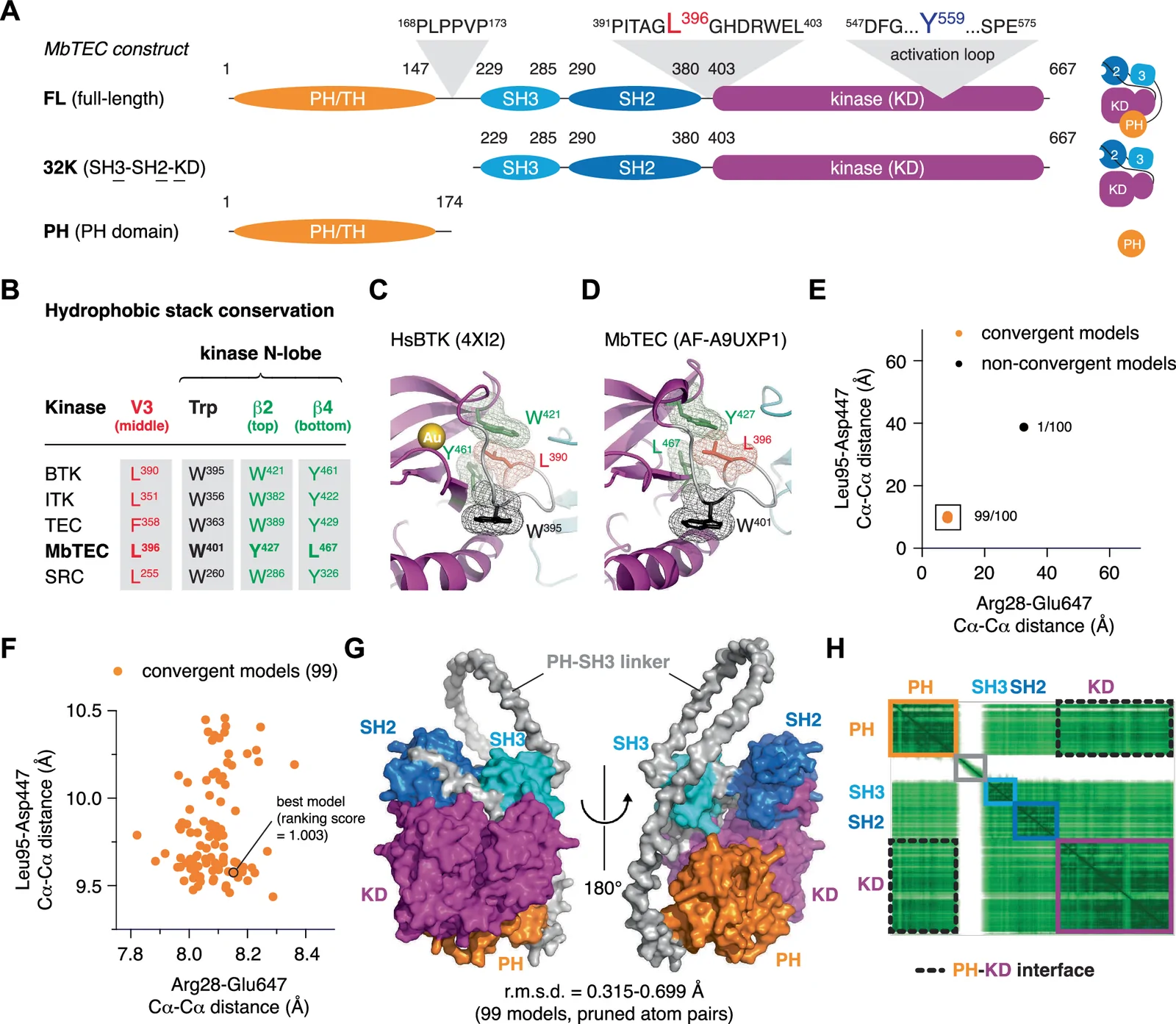
*M. brevicollis* TEC adopts a conserved autoinhibited conformation. **A** Domain schematics of all protein constructs used in this study. **B** Hydrophobic stack conservation in the TEC kinases. **C** Structure of hydrophobic stack of mouse BTK. The middle residue in the stack, contributed by the SH2-kinase linker, is shown in red. The three residues in the stack are shown in mesh representation. **D** AlphaFold-predicted structure of MbTEC hydrophobic stack. The three residues in the stack are shown in mesh representation. **E** Euclidean distance plot of two PH-kinase domain residue pairs in 100 models generated by AlphaFold 3 from a curated, taxonomically balanced MSA. Inter-residue Cα–Cα distances for Arg28-Glu647 and Leu95-Asp447 provide an indicator of model convergence. **F** Zoomed-in plot of the 99 convergent models shown in the black box in (**E**). The best ranked model is indicated with a black outline and its AlphaFold 3 ranking score. **G** Overall structure of the best ranked model and root mean square deviation over pruned atom pairs between the 99 models. **H** Pair alignment error plot for the best ranked model. Individual domains are depicted with colored rectangles according to the color scheme indicated in (**A**). Black, dashed rectangles indicate the regions of the plot that correspond to residue pairs between the PH and kinase domains.

MbTEC is a 667 amino acid long protein with 42% sequence identity to human BTK. It comprises a PH domain at its N-terminus followed immediately by a TEC homology (TH) domain, an unstructured linker of approximately 85 amino acids that contains a conserved polyproline motif, SH3, SH2, and kinase domains (Fig. 1A). The TH domain is not an independently folding domain, but a zinc finger that is an intrinsic part of the PH domain fold^16^. MbTEC Y559 in the activation loop corresponds to the canonical autophosphorylation site (human BTK Y551). A conserved hydrophobic amino acid in the SH2-kinase linker (MbTEC L396) forms the central component of a three-residue hydrophobic stack (Fig. 1B–D and Supplementary Fig. 1A) that serves to maintain the SH3-SH2-kinase module of SFKs in an inactive conformation stabilized by ADP^29^. Mutation of the central residue in the hydrophobic stack is sufficient to convert both SRC and ITK into an active conformation, concomitant with the binding of ATP^29^.

To study the properties of MbTEC, we purified wild-type and mutant variants of the full-length protein as well as its isolated PH domain and 32K modules (Fig. 1A) to homogeneity. All kinase domain-containing constructs were dephosphorylated in vitro with the tyrosine phosphatase YopH and the serine/threonine lambda phosphatase after affinity purification, prior to ion exchange and size-exclusion chromatography (SEC). The mass and polydispersity of all purified proteins employed in this study were confirmed by mass spectrometry (Supplementary Fig. 2A–N) and mass photometry (Supplementary Fig. 3A–L) or SEC coupled to multi-angle light scattering (SEC-MALS) (Supplementary Fig. 3C). To investigate the nucleotide dependence of MbTEC, we measured its thermal stability in the presence of ADP, ATP, and a non-hydrolyzable ATP analog, AMPPNP. MbTEC was preferentially stabilized by ADP (Supplementary Fig. 4A), consistent with what has previously been observed for SRC and ITK^29^.

Advances in computational modeling have made the determination of proteins of unknown structure routine^30^, including, recently, the docking of ligands and ions in AlphaFold 3^31^. Using a standard implementation of AlphaFold 3, we observed consistent model convergence on the known intramolecular architecture of the SH3, SH2, and kinase domains, but divergent placement of the PH domain (Supplementary Fig. 4B). Suspecting that AlphaFold may be failing to detect a co-evolutionary signal for the PH–kinase interface in the multiple sequence alignment (MSA) used for structure inference, we investigated the quality of the MSA. AlphaFold 3 uses JackHMMER^32^ to detect remote sequence homologs of protein domains using profile hidden Markov models such that evolutionary sequence depth provides strong couplings that guide de novo structure inference of the folded domains^32^. However, an MSA that contains partially homologous sequences that do not belong to the same family will dilute physiologically relevant evolutionary couplings between the domains of one protein. The MSA generated by AlphaFold 3 contained 50,000 sequences, of which 37,072 were found to be unique. Using InterProScan, we screened each sequence for the presence or absence of each of the functional domains of MbTec (Supplementary Fig. 4C). Of the 37,072 unique sequences, only 1898 (3.7%) corresponded to full-length sequences containing all 5 domains (PH, TH, SH3, SH2, KD). These observations indicate that, without subsampling, the default MSA generated by AlphaFold is unsuitable for structure inference of a full-length Tec kinase, specifically with respect to intramolecular domain-domain interactions.

To address these problems, we curated a set of 2442 TEC kinase sequences by filtering UniProt for the presence of PH, Btk_Zn_finger, SH3, SH2, and tyrosine kinase domains as defined by InterPro^33^ (Supplementary Fig. 4C). After aligning the sequences using MAFFT^34,35^, we corrected for taxonomic bias by reweighting or downsampling (Supplementary Methods and Supplementary Fig. 5A) and fed the resulting MSAs into AlphaFold 3. To determine model convergence, we selected two pairs of residues between each of the regulatory PH, SH3, and SH2 domains and the kinase domain and plotted the Euclidean distances between the Cα atoms of each pair of residues (Supplementary Fig. 5B–F). In the standard AlphaFold 3 pipeline without MSA curation, 39% model convergence with an identical PH–kinase domain interface was obtained when including a single Zn^2+^ ion (Supplementary Fig. 5C), which is present in an experimentally determined structure of the PH domain in BTK^16^. Structure prediction with our curated MSA resulted in a modest increase in model convergence to 52% (Supplementary Fig. 5A), while taxonomic reweighting of the sequences increased this figure to 80% (Supplementary Fig. 5A) and taxonomic downsampling increased this further to 99% (Fig. 1E, F and Supplementary Fig. 5A, D–F). The resulting MbTEC models exhibit a conserved 32K architecture superimposable with the crystal structure of the 32K module of human Btk^6^ and the ADP-bound conformation of Src^29^, as well as structures of Hck^8^ and Abl^10^ (Supplementary Fig. 5I, J). All models capture the inactive conformation of the activation loop tyrosine, which is sequestered in a non-phosphorylatable position. Finally, the pair-alignment error (PAE) plot for the highest-scoring (AlphaFold 3 ranking score) model (Fig. 1F, G) exhibits strong residue couplings between the PH and kinase domains (Fig. 1H). These findings imply an evolutionarily conserved interaction between PH and kinase domains in the TEC kinases.

### MbTEC adopts a compact conformation in solution

**Fig. 2:**
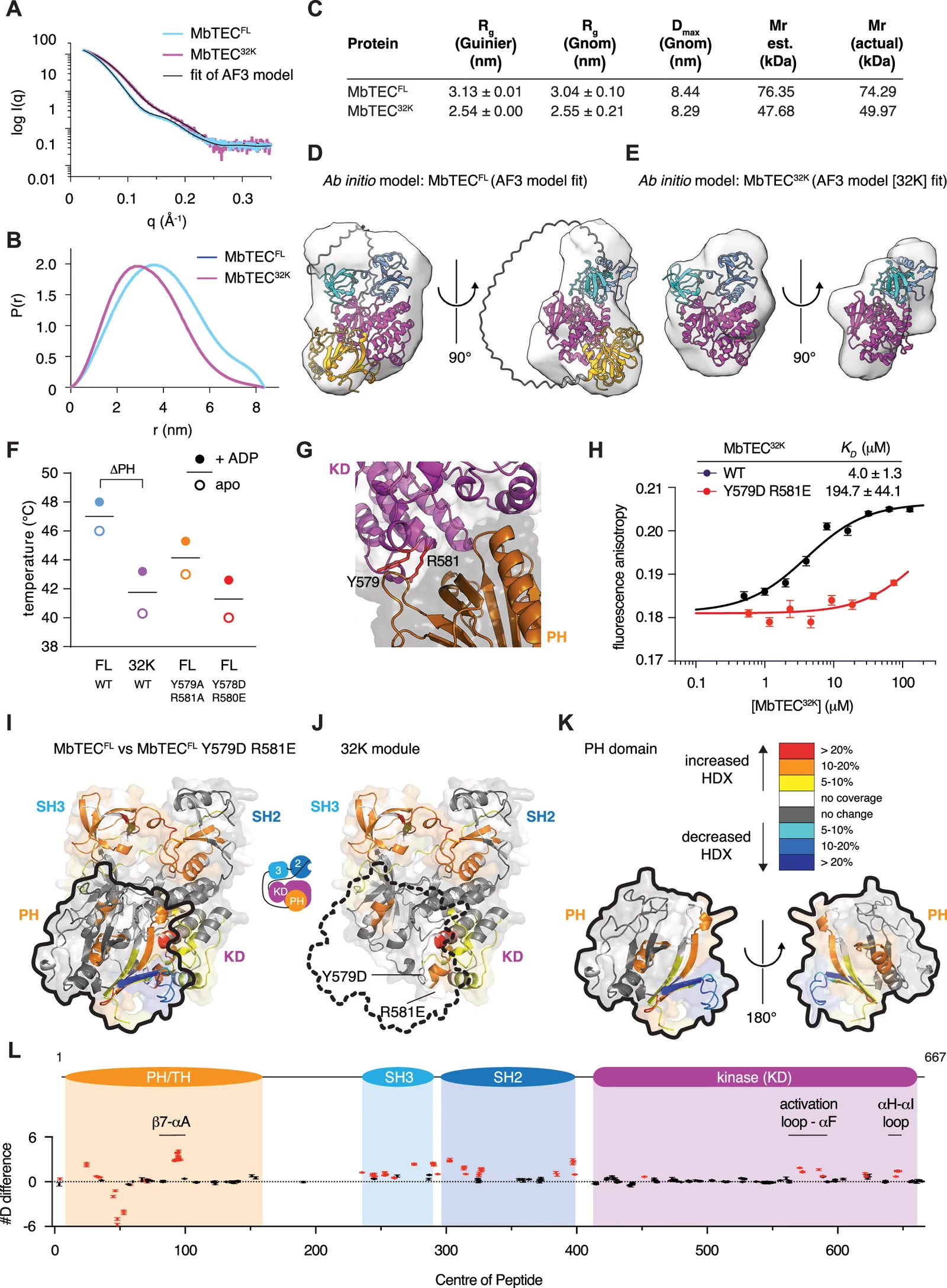
MbTEC adopts a compact conformation in solution. **A** Small-angle X-ray scattering (SAXS) curves of MbTEC^FL^ and MbTEC^32K^ in solution. The theoretical scattering curves of the AlphaFold models of MbTEC^FL^ and MbTEC^32K^ and the fits to their respective scattering curves are shown as solid lines. **B** Pair distribution functions for MbTEC^FL^ and MbTEC^32K^. **C** Summary table of particle parameters derived from SAXS measurements. **D** Ab initio molecular modeling of the molecular envelope of MbTEC^FL^. The AlphaFold model of MbTEC^FL^ was docked into the envelope using SUPCOMB^81^. **E** Ab initio molecular modeling of the molecular envelope of MbTEC^32K^. The AlphaFold model of MbTEC^32K^ was superimposed with the model using SUPCOMB^81^. **F** Melting temperatures of MbTEC^FL^ (blue), MbTEC^32K^ (purple), MbTEC^FL^ Y579A R581A (orange), and MbTEC^FL^ Y579D R581E (red) with (filled circle) or without (empty circle) ADP. **G** Location of Y579 and R581 in the PH–kinase domain interface. **H** Binding of MbTEC^32K^ (WT) and MbTEC^32K^ Y579D R581E to MbTEC^PH^ determined by fluorescence anisotropy. Each data point is the average of ten measurements. The data were fit with a one-site binding model to determine *K*_D_. **I** Pairwise HDX-MS analysis of MbTEC^FL^ compared to MbTEC^FL^ Y579D R581E. Significant differences in HDX in MbTEC^FL^ Y579D R581E are mapped onto the AlphaFold3 model of MbTEC^FL^ and are color coded according to the legend. The PH domain is outlined with a black stroke. **J** HDX changes mapped on the 32K module of MbTEC (PH domain cut away). The surface of the PH domain is outlined with a dashed stroke. The locations of Y579 and R581 are indicated on the kinase domain. **K** HDX changes mapped on the PH domain of MbTEC. **L** Sum of #D difference in deuterium incorporation over the entire time course. Each point represents an individual peptide with significant changes shown in red (>0.40 Da, 5% difference, and two-tailed *t* test *p* < 0.01 at any timepoint, *n* = 3). Domain architecture is annotated above.

To characterize the size, shape and conformation of MbTEC in solution, we employed SEC coupled to small-angle X-ray scattering (SEC-SAXS). Frames were averaged across the elution peak and reduced to a 1D scattering curve (Fig. 2A). Guinier plots for MbTEC^FL^ and MbTEC^32K^ are both linear in the low-angle regime (Supplementary Fig. 6A, B), indicating that the radius of gyration is well estimated in each case. From the pair distribution functions (Fig. 2B), MbTEC^FL^ has an *R*_g_ of 3.1 nm and a maximum dimension of 8.4 nm compared with 2.5 nm and 8.3 nm for MbTEC^32K^ (Fig. 2C). Estimation of the molecular mass from the scattering is within 2 kDa of the actual mass of the proteins (Fig. 2C). Calculation of the theoretical solution scattering of our AlphaFold 3 model for the autoinhibited conformation of MbTEC indicated a remarkable fit to the experimental scattering, both for MbTEC^FL^ and MbTEC^32K^ (Fig. 2A). Ab initio calculation of the respective molecular envelopes from the scattering curves and fitting of the respective models for MbTEC^FL^ (Fig. 2D) and MbTEC^32K^ (Fig. 2E) also revealed a strong correlation between model and experiment. This suggests MbTEC forms a compact conformation in solution that is well described by the AlphaFold model in Fig. 1G.

Consistent with the loss of an energetically favorable interface, deletion of the PH domain resulted in a 6 °C reduction in thermal stability (Fig. 2F and Supplementary Fig. 6C). We next tested the specificity of the predicted PH-kinase interaction by mutating Y579 and R581, which are conserved residues in the interface (Fig. 2G). Mutation of Y579 and R581 to alanine reduced thermal stability by 3 °C, while their mutation to aspartate and glutamate, respectively, resulted in the same thermal stability as MbTEC^32K^ lacking its PH domain (Fig. 2F and Supplementary Fig. 6D). These observations indicate that substitution of Y579 and R581 with alanine weakens the autoinhibitory conformation by reducing van der Waals contacts, but substitution with charged residues that introduce unfavorable interactions is sufficient to completely disrupt the interface. Consistently, MbTEC^32K^ bound to the PH domain with an affinity of 4.0 μM, but binding of MbTEC^32K^ Y579D R581E was barely detected (Fig. 2H).

To map the intramolecular association of the MbTEC PH and kinase domains experimentally, we employed HDX-MS. HDX-MS provides peptide-level information on the rate of exchange of backbone amide hydrogens with a deuterated solvent, thereby revealing changes in secondary structure when comparing two species. To investigate the surfaces of MbTEC involved in PH domain sequestration, we compared the rates of HDX between MbTEC^FL^ and MbTEC^FL^ Y579D R581E. Mutation of the interface resulted in significant increases in the rate of HDX throughout the SH3 domain, SH2 domain, PH domain and in the αC helix, activation loop, and αH-αI loop of the kinase domain (Fig. 2I–L). There was a single region in the PH domain that showed decreased exchange in the mutant. These changes could be recapitulated by deletion of the PH domain (Supplementary Fig. 6E–H), HDX increases similar to those previously reported for the same comparison in human BTK^20^. The αC helix, activation loop, and αH-αI loop are all predicted to be at least partially sequestered in interactions with the PH domain. This data also suggests a role of the PH domain in stabilizing inhibitory contacts of the SH2 and SH3 domains. The time course of deuterium incorporation is shown for individual peptides in these regions in Supplementary Fig. 6I (MbTEC^FL^ vs MbTEC^32K^) and Supplementary Fig. 6J (MbTEC^FL^ vs MbTEC^PH^), with the full set of deuterium incorporation data shown in the source data.

### MbTEC is autoinhibited by its PH domain

**Fig. 3:**
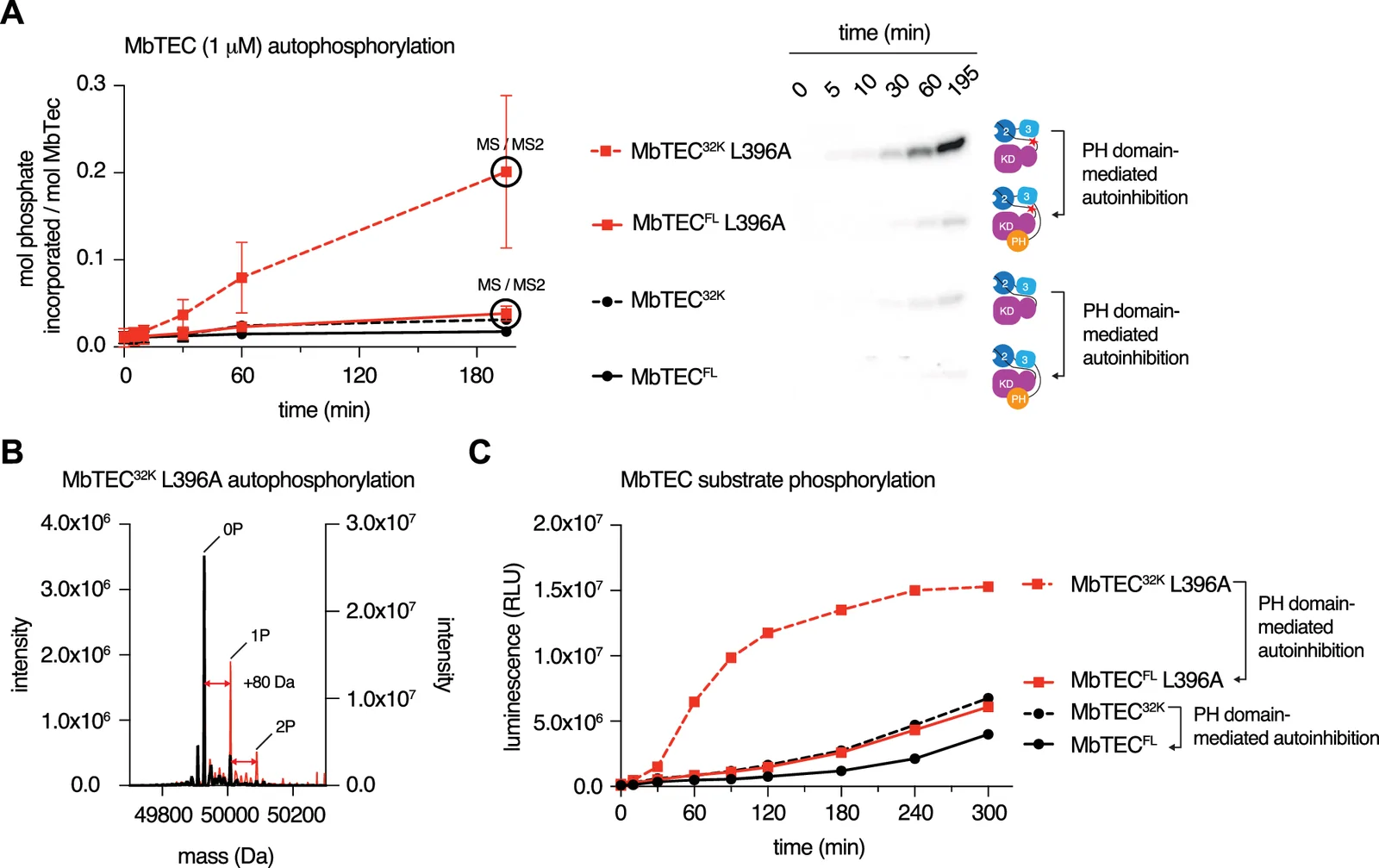
MbTEC is autoinhibited by its PH domain. **A** Radiometric autophosphorylation assays of MbTEC^FL^ (black, solid line) and MbTEC^32K^ (black, dashed line), as well as their L396A counterparts (red). Right: representative cartoons and autoradiograph for each construct. **B** Mass spectrometry analysis of the product of MbTEC^32K^ L396A autophosphorylation (denoted by the black circle in (**A**)). Intensities before autophosphorylation in black (left axis) and after autophosphorylation in red (right axis). **C** Substrate peptide phosphorylation by MbTEC^FL^ (black, solid line), MbTEC^FL^ L396A (black, dashed line), MbTEC^32K^ (red, solid line), and MbTEC^32K^ L396A (red, dashed line). ADP-Glo kinase assay; RLU (raw luminescence units).

Although the activation of TEC kinases by PIP_3_-containing membranes has previously been demonstrated^6,7,14,21,26^, these experiments cannot deconvolute the relative influences of local concentration and allosteric effects on TEC activation. Since autophosphorylation depends on dimerization, the local concentration of a TEC kinase on a membrane will necessarily result in its activation, regardless of whether there is an allosteric component. Other studies, however, have reported an increase in BTK and ITK activity upon deletion of the PH domain^6,22^ as well as conformational changes in BTK upon PIP_3_ binding^26^, indicating that the PH domain exerts an autoinhibitory effect in the absence of PIP_3_. To determine whether the PH domain exerts an autoinhibitory effect on MbTEC, we compared the rates of autophosphorylation of MbTEC^FL^, MbTEC^FL^ L396A, MbTEC^32K^, and MbTEC^32K^ L396A using a radiometric kinase assay (Fig. 3A). MbTEC^FL^ exhibited the lowest rate of autophosphorylation, which was only modestly increased by either deletion of the PH domain or mutation of L396 in the SH2-kinase linker, a mutation known to promote the constitutive activation of ITK^29^. Combining deletion of the PH domain with mutation of L396, however, increased the rate of autophosphorylation more than tenfold (Fig. 3A). These observations indicate that the PH domain exerts a strong autoinhibitory influence on kinase activity that cannot be overcome by disruption of the SH3-kinase interaction. These observations indicate that the PH and SH3 domains exert synergistic inhibitory effects on the kinase domain and that disengagement of both domains by ligand binding is required for complete activation of MbTec. This is the equivalent of an AND gate in an electronic circuit, as opposed to an OR gate.

To assess the stoichiometry of autophosphorylation of MbTEC, we measured the mass of MbTEC^FL^ L396A and MbTEC^32K^ L396A pre- and post-autophosphorylation by mass spectrometry. We analyzed samples in the linear kinetics regime to capture the most specific events. The increase in mass corresponded to modification with a single phosphate in 40% of MbTEC^32K^ L396A molecules under the conditions of the assay (Fig. 3B). By contrast, MbTEC^FL^ L396A exhibited negligible autophosphorylation (Supplementary Fig. 7A), consistent with robust autoinhibition by its PH domain. To identify the modified residue, MbTEC^32K^ L396A and MbTEC^FL^ L396A were subjected to tandem mass spectrometry, which revealed phosphorylation of the canonical activation loop tyrosine Y559 in MbTEC^32K^ L396A, but not MbTEC^FL^ L396A (Supplementary Fig. 7B). This confirmed that the PH domain specifically inhibits activation loop phosphorylation of MbTEC.

Finally, we observed that phosphorylation of a consensus substrate peptide is also robustly activated by the additive effects of both PH and SH3 domain displacement (Fig. 3C). Together, these observations indicate that MbTEC is robustly autoinhibited by its PH domain and that PH domain displacement is necessary for its activation.

### A gain-of-function mutation in the PH domain activates MbTEC

**Fig. 4:**
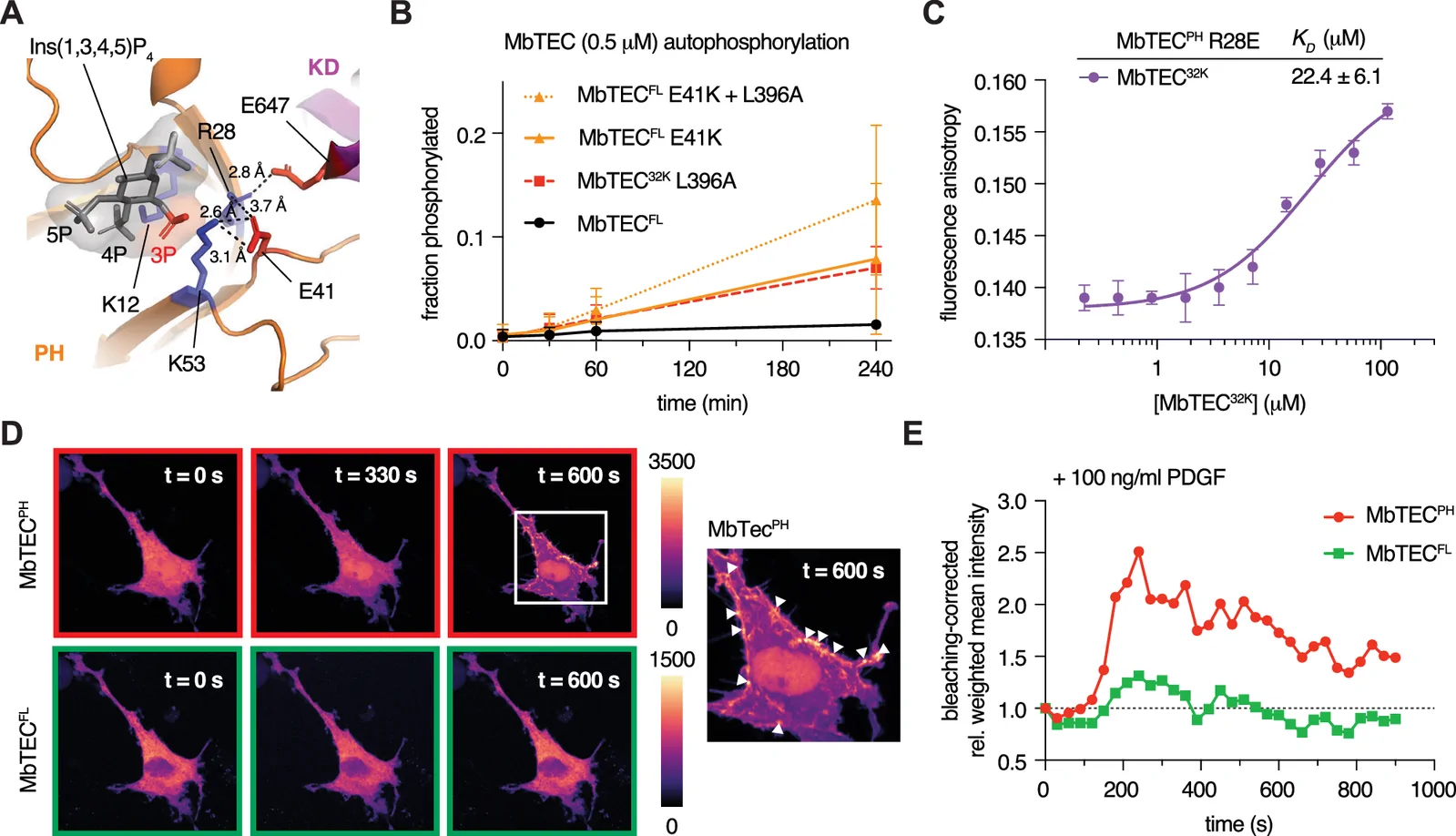
A gain-of-function mutation in the PH domain activates MbTEC. **A** High-resolution view of the PIP_3_ binding site in the context of autoinhibited MbTEC. Residues that contribute to both PIP_3_ recognition and stabilization of the PH–kinase interface are depicted in sticks colored according to their acidic (red) or basic (blue) character. The head group of PIP_3_ was positioned in the model by superimposing the structure of the BTK PH domain in complex with Ins(1,3,4,5)P_4_ (PDB: 1B55). The 3’ phosphate crucial for TEC activation is colored red. Hydrogen bonds are indicated with dashed lines. **B** Radiometric autophosphorylation assays of MbTEC^FL^ (black, solid line) and MbTEC^FL^ E41K (orange, solid line), as well as MbTEC^32K^ L396A (red, dashed line) and MbTEC^FL^ E41K L396A (orange, dashed line). Measurements are the average of three independent replicates. **C** Binding of MbTEC^32K^ (wt) to MbTEC^PH^ (R28E) determined by fluorescence anisotropy. Each data point is the average of 10 measurements. The data were fit with a one-site binding model to determine *K*_D_. **D** Representative images of the membrane translocation of MbTEC^FL^ (green channel) versus MbTEC^PH^ (red channel) over a time course of 10 min in co-transfected, PDGFαβ-treated NIH3T3 fibroblasts. Right: lookup tables (LUT) representing the color-encoded intensity map of individual pixels. White arrowheads: membrane foci of enriched MbTEC^PH^. **E** Bleaching-corrected, normalized mask intensity profiles of MbTEC^FL^ (green) and MbTEC^PH^ (red) in a representative cell as a function of time, post-PDGFαβ stimulation.

A previously identified gain-of-function mutation in the PH domain of BTK, E41K, has been proposed to activate BTK by enhancing its binding to PIP_3_ at the plasma membrane^17,18,36^ as well as promoting IP_6_-mediated dimerization^37^. Interestingly, E41 is predicted to make hydrogen bonds to R28 and K53 in the autoinhibited conformation of MbTEC (Fig. 4A), where R28 is also stabilized by a hydrogen bond to E647 in the kinase domain. As such, the E41K mutation would be expected to destabilize the PH–kinase interface by introducing charge repulsion with R28 and K53. Consistent with this hypothesis, MbTEC^FL^ E41K (equivalent to human BTK E41K) exhibited a significantly increased rate of autophosphorylation in the absence of PIP_3_ compared to wild-type MbTEC^FL^, equivalent to the rate of autophosphorylation of MbTEC^32K^ L396A, indicating that the E41K mutation weakens PH domain-mediated autoinhibition (Fig. 4B). The kinetics of MbTEC^FL^ E41K autophosphorylation could be further enhanced by introduction of the L396A mutation, simulating SH3 domain release (Fig. 4B). Although R29X in human BTK is a loss-of-function mutation in XLA due to a loss of PIP_3_ binding and a consequent failure to activate BTK downstream of the BCR^16,17,38^, mutation of MbTEC^PH^ R28 to glutamate also weakened the PH–kinase interface by fivefold (Fig. 4C), consistent with the sequestration of the PIP_3_ binding pocket predicted by our model.

Partial sequestration of the PIP_3_-binding surface by the kinase domain implies that there is a competition between PIP_3_ and the kinase domain for binding to the PH domain. The logical consequence of this should be a reduced affinity for PIP_3_ in full-length MbTEC and, conversely, enhanced binding by the isolated PH domain. To test this hypothesis in cells, we compared the membrane translocation behavior of co-expressed MbTEC^FL^ with MbTEC^PH^ within individual growth factor-stimulated fibroblasts. MbTEC^FL^ exhibited a primarily diffuse cytoplasmic distribution with minor enrichment of MbTEC^PH^ at membrane protrusions (Fig. 4D). Upon stimulation of the cells with PDGFαβ, MbTEC^PH^ exhibited considerably stronger enrichment at membrane protrusions in comparison to MbTEC^FL^ (Supplementary Fig. 6A). Importantly, while the membrane association of MbTEC^FL^ was transient, MbTEC^PH^ exhibited prolonged association with the membrane long after MbTEC^FL^ had returned to its basal level post-stimulation of the cells with PDGFαβ (Fig. 4E and Supplementary Fig. 8B). Together, these observations support an autoinhibitory role for the PH domain in which the PIP_3_-binding pocket is partially sequestered in the interface and PIP_3_ elicits MbTEC activation by destabilizing a network of electrostatic interactions between the PH domain and the kinase domain.

### MbTEC is allosterically activated by PIP_3_

**Fig. 5:**
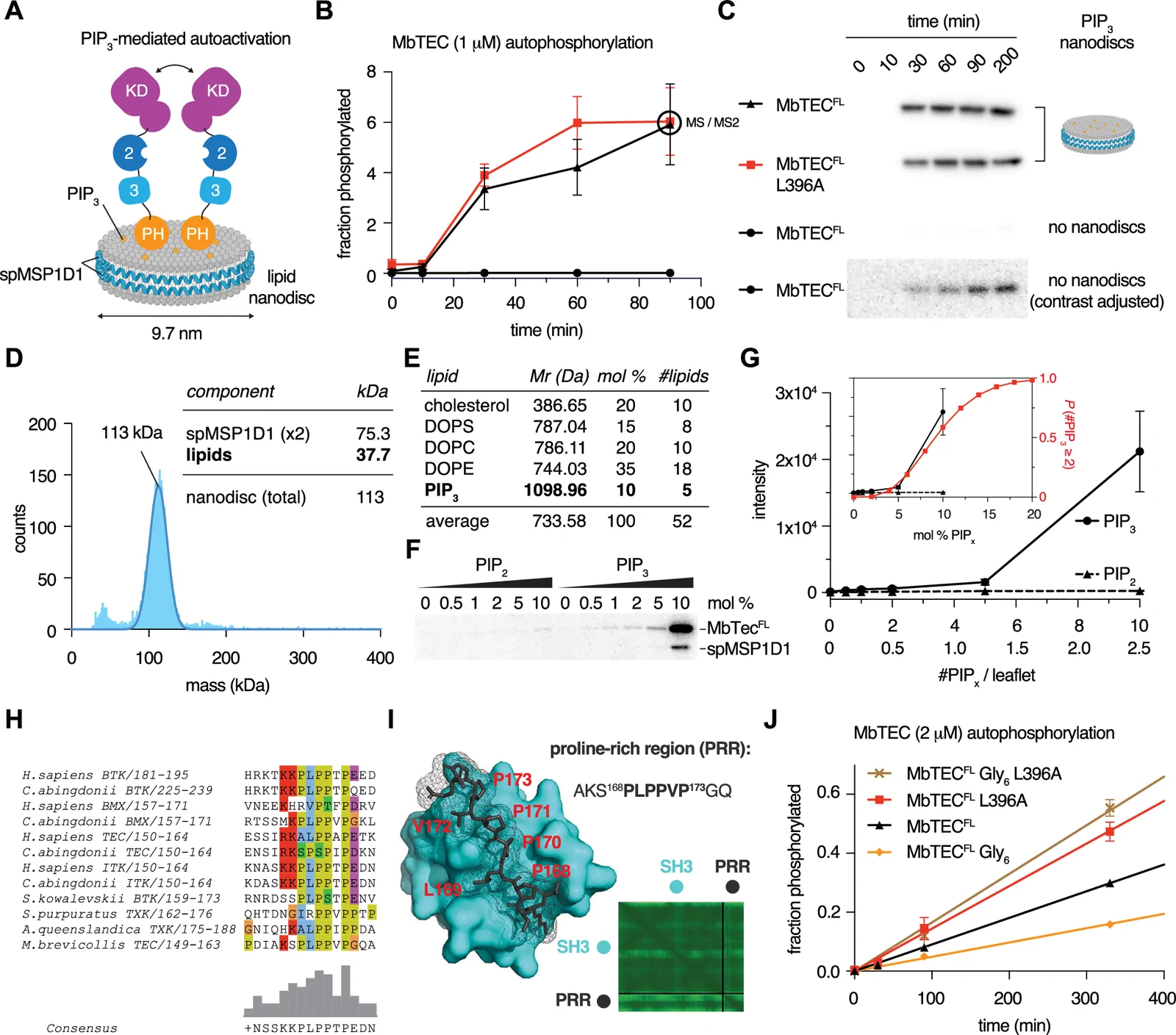
MbTEC is allosterically activated by PIP_3_. **A** PIP_3_-doped nanodiscs used to activate MbTEC. MSP1D1 membrane scaffold protein. **B** PIP_3_-mediated activation of MbTEC^FL^ (black, triangles) and MbTEC^FL^ L396A (red, squares). Black, circles: MbTEC^FL^ without PIP_3_ nanodiscs. **C** Representative autoradiographs for each construct quantified in (**B**). Note: the contrast has been enhanced for the purposes of displaying the low basal autophosphorylation of MbTEC^FL^ in the absence of PIP_3_ nanodiscs. 200 min time points were not quantified in (**B**) due to saturation of the phosphor screen. **D** Mass photometry of PIP_3_-doped MSP1D1 nanodiscs. Inset table: molecular mass of MSP1D1 and lipid components. **E** Lipid composition of nanodiscs in mole percentages and average number of lipids per nanodisc. **F** Representative autoradiograph of MbTEC autophosphorylation in the presence of lipid nanodiscs containing increasing concentrations of PIP_2_ or PIP_3_. **G** Quantification of the experiment represented in (**F**) (*n* = 3). Inset plot: overlay of experimental data (black) with the cumulative probability distribution (red) of finding four PIP_3_ molecules per nanodisc (two per membrane leaflet) at each PIP_3_ concentration. **H** Sequence conservation of the polyproline motif between TEC paralogs and over the course of vertebrate evolution. Representative human and turtle (*C. abingdonii*) sequences. **I** AlphaFold prediction of the intramolecular association of the TEC kinase polyproline motif with its SH3 domain. **J** Radiometric autophosphorylation time course of MbTEC^FL^ (black) and MbTEC^FL^ L396A (red), as well as MbTEC^FL^ Gly_6_ (orange) and MbTEC^FL^ Gly_6_ L396A (brown).

Structurally and biochemically, the evidence presented so far strongly supports the allosteric activation of MbTEC by PIP_3_. To demonstrate it directly, we employed PIP_3_-containing lipid nanodiscs (Fig. 5A). We observed that the rate of MbTEC autophosphorylation is dramatically enhanced by PIP_3_ binding (Fig. 5B, C). Mass spectrometry revealed autophosphorylation of up to eight sites in MbTEC elicited by PIP_3_ (Supplementary Fig. 9A), of which five sites correspond to tyrosines in the SH3 and SH2 domains (Supplementary Fig. 9B, C), as well as *trans*-phosphorylation of the nanodisc membrane scaffold protein (MSP1D1) (Supplementary Fig. 9D). These observations indicate that additional interactions within the “signalosome” are likely required for high-fidelity auto- and substrate phosphorylation. To confirm that MbTec is specifically activated by PIP_3_, we measured autophosphorylation in the presence of nanodiscs containing increasing concentrations of PIP_2_ or PIP_3_. We measured the mass of the nanodiscs by mass photometry (Fig. 5D), from which we calculated the average number of lipids per nanodisc for each component (Fig. 5E). MbTec was specifically and robustly activated by PIP_3_, but not PIP_2_ (Fig. 5F, G). Interestingly, MbTec autophosphorylation tracks with the probability of finding four or more PIP_3_ molecules per nanodisc (≥2 molecules/membrane leaflet) (Fig. 5G, inset, red curve), suggesting that autophosphorylation depends critically on the coincident allosteric activation of two molecules of MbTec by PIP_3_.

Curiously, an additive effect of PIP_3_ binding and displacement of the SH3 domain (L396A) was not manifested in increased kinetics of autophosphorylation (Fig. 5B, C), as previously observed when the PH domain was deleted (Fig. 3A). This finding suggests that PIP_3_ binding elicits complete allosteric activation of MbTEC^FL^, which cannot be phenocopied by simple deletion of the PH domain in MbTEC^32K^. Previous studies have reported an intramolecular association of the SH3 domain with a conserved proline-rich region (PRR) in the PH-SH3 interdomain linker^39–41^ (Figs. 1A and 5H), while a mutagenesis study has implicated the PRR in the activation of BTK^26^. As proposed for BTK, the intramolecular association of the SH3 domain with this motif upon the allosteric activation of MbTEC by PIP_3_ is supported by AlphaFold predictions of the interface that converge on near-identical, high-confidence models (Fig. 5I). These structures are superimposable with experimentally determined structures of the SH3 domain associated with the polyproline motif of both ITK^39^ and TEC^40^. To test whether the polyproline motif contributes to MbTEC activation in the absence of adapter proteins, we compared the autophosphorylation kinetics of wild-type MbTEC^FL^ with MbTEC^FL^ bearing a mutated PRR motif (MbTEC^FL^ Gly_6_) (Fig. 5J). We observed that MbTEC^FL^ Gly_6_ autophosphorylated considerably slower than MbTEC^FL^, suggesting that the polyproline motif plays an active role in MbTEC activation. A similar decrease in autophosphorylation was previously reported for human BTK mutated in its PRR^26^. As observed previously, mutation of L396 in the SH2-kinase linker activated MbTEC, but addition of the PRR mutation (MbTEC^FL^ Gly_6_ L396A) did not have any additive effect. This finding is consistent with the situation in which the polyproline motif can compete with the SH2-kinase linker for binding to the SH3 domain under conditions in which autoinhibition by the PH domain is relieved.

In summary, activation of MbTEC is explicitly dependent on PIP_3_, which triggers displacement of the PH domain and subsequent engagement of the PRR by the SH3 domain, leading to relief of autoinhibition, autophosphorylation, and downstream substrate phosphorylation.

## Discussion

This study describes the activation of a TEC kinase by the lipid second messenger PIP_3_ with unprecedented mechanistic insight. Using a custom in silico pipeline for structure prediction with AlphaFold 3, we describe the nature of the autoinhibitory interface between the PIP_3_-binding PH domain and the kinase domain at atomic resolution. We validate the model with in-solution X-ray scattering data and HDX-MS analysis of truncation and interface mutants, and demonstrate a specific, physical interaction of the PH and kinase domains in *trans*. Finally, we present activity data supporting a model in which PIP_3_, but not PIP_2_, elicits the allosteric activation of MbTEC by destabilizing an electrostatic network of inhibitory interactions between the PH and kinase domains. We find that autophosphorylation of MbTec elicited by PIP_3_ corresponds to the probability of finding two or more PIP_3_ molecules in a single leaflet of the membrane bilayer, consistent with the known dependency of autophosphorylation on dimerization^14,19^. Our findings converge on a model for the activation of MbTEC by PIP_3_ that is likely shared by all TEC kinases.

Although PIP_3_ signaling has not been explicitly demonstrated in a choanoflagellate, the machineries for its production predate choanoflagellates by at least 500 My^42^. PI3K-mediated production, PH domain-mediated sensing, and PTEN-mediated degradation of PIP_3_ have all been robustly demonstrated to control chemotaxis in the slime mold *Dictyostelium discoideum*^43–48^. While the Tec kinases emerged more recently^5^, PI3K, PTEN, PDK1, and Akt are all found in choanoflagellates, suggesting that choanoflagellates inherited an ancient lipid signaling toolkit and that the Tec kinases were a novel evolutionary addition to the toolbox.

In addition to elucidating the mechanism of PIP_3_-mediated activation of the Tec kinases, our work has important implications for the accurate structure prediction of proteins and protein complexes. AlphaFold exploits deep co-evolutionary variance to predict protein structure. At its core is a MSA typically generated by profile hidden Markov models that can detect remote homologs. While this is a robust tool for predicting the fold of individual domains, it risks contaminating the MSA with partially homologous, but divergent sequences that mask evolutionary couplings between residues. Greater emphasis, therefore, needs to be placed on MSA curation to ensure that the input sequences belong to genuine protein orthologs. Similarly, our work emphasizes the risk of taxonomic bias in the MSA, something which AlphaFold does not explicitly address. Taxonomic bias arises from the over-representation of highly similar sequences and can lead to weaker signals for co-evolutionary variance. By addressing both of these issues, we were able to confidently predict the structure of full-length MbTEC where the standard AlphaFold implementation fails. While the importance of MSA curation, clustering, sampling and taxonomic balancing have all been previously reported^49–53^, they have yet to be more widely adopted by the vast community of scientists employing AlphaFold in their studies.

A clear extension of these insights is that proper curation of an MSA will likely give rise to structural models that have a deep evolutionary conservation. In this case, the convergence on a singular model for the autoinhibited conformation of MbTEC implies that this conformation is conserved among all the TEC kinases. Further experimentation will no doubt be required to confirm this assumption, but literature evidence suggests that the model is compatible with the solution conformations of human ITK^21^ and BTK revealed by NMR and HDX-MS, respectively^20^, meaning that we have already spanned the approximately 600–800 My of evolution between choanoflagellates and humans.

**Fig. 6:**
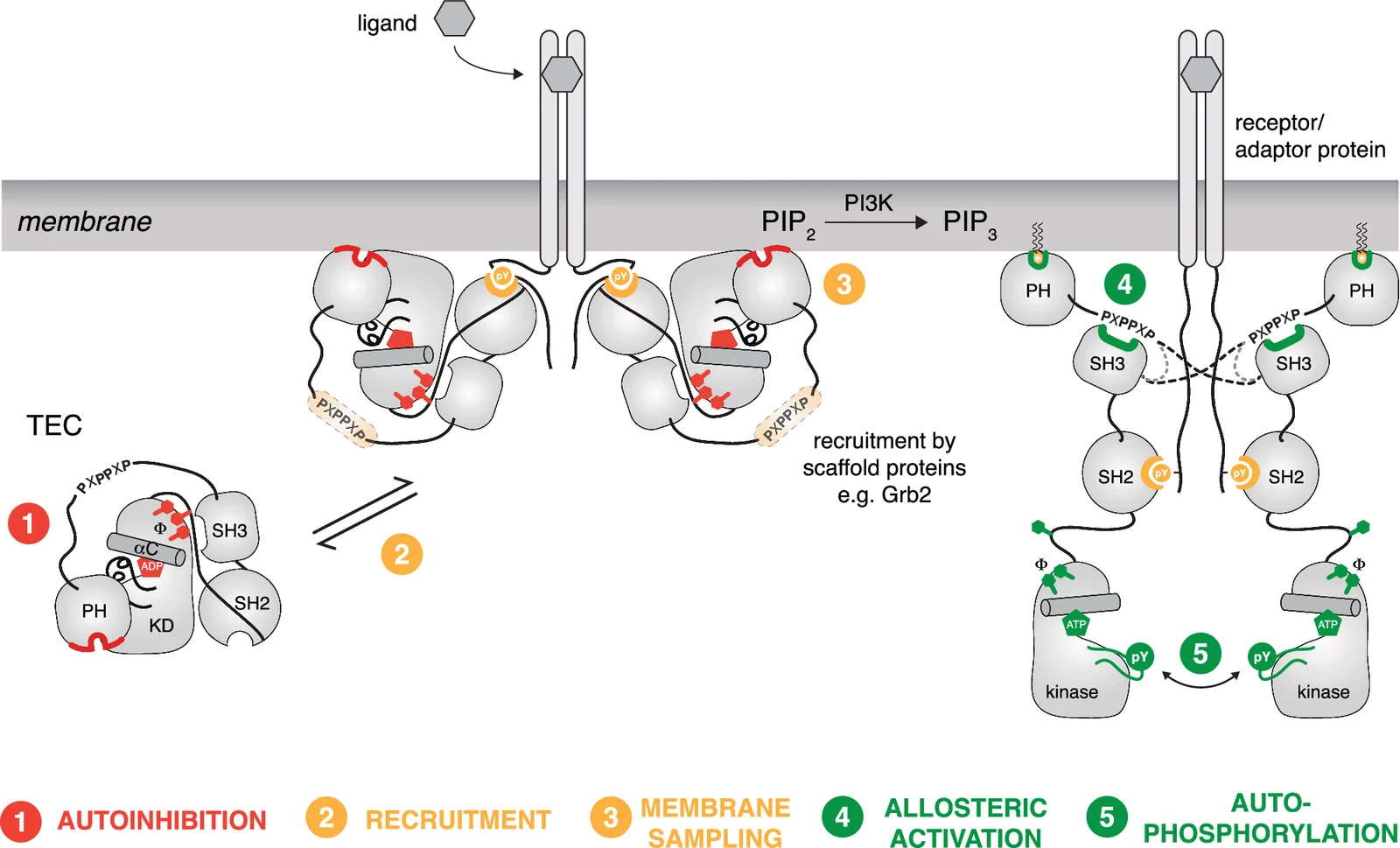
Model of PIP_3_-mediated auto-activation of TEC family kinases. TEC kinases adopt an autoinhibited conformation in the absence of an activating stimulus that is characterized by a discrete inhibitory interface between its PH and kinase domains (1). Upon ligand-mediated receptor activation and subsequent phosphorylation by non-RTKs, TEC is recruited to the membrane by phospho-tyrosine motifs in the receptor tails and SH3 domain-containing adapter proteins, such as Grb2 (2). Local concentration of TEC beneath the plasma membrane permits sampling of the membrane for PIP_3_ (3), engagement of which is concomitant with disengagement of its PH and SH3 domains from its kinase domain. PIP_3_ binding anchors TEC on the membrane and licenses its SH3 domain to compete with Grb2 for the polyproline motif in the PH-SH3 linker, thereby promoting allosteric activation of the kinase domain (4). Dimerization of TEC, driven by its local concentration on the membrane and possibly by reciprocal binding of the SH3 domain to the polyproline motif of a second TEC protomer, promotes activation loop autophosphorylation (5), thereby rendering TEC fully competent for downstream signaling. Upon loss of the PIP_3_ signal, TEC is autoinhibited by its PH domain, thereby eliciting an acute termination of downstream signaling.

All of this leads us to propose a universal model for the activation of the TEC kinases by PIP_3_. Activation of cell surface receptors drives phosphorylation of their cytoplasmic tails by non-receptor tyrosine kinases (Fig. 6). These phospho-tyrosine marks serve to recruit both the TEC kinase and class I PI3K. Recruitment of TEC alone is insufficient to drive its activation, which depends on the production of PIP_3_ by PI3K. Co-localization of autoinhibited TEC and PI3K at the plasma membrane increases the probability that TEC will be activated by PIP_3_ upon its production; indeed, XLA-associated mutations of the SH2 domain of BTK, including the phospho-tyrosine coordinating residue, R307, inhibit binding to the adapter protein B cell linker protein^54,55^ and impair BTK activation in cells^25^. The SH2 domain of ITK is similarly required for its activation downstream of the TCR^56^. While PI(4,5)P_2_ can bind to the PIP_3_ binding pocket of the PH domain, it is the specific recognition of the 3’ phosphate by residues K12, R28, and K53 that weakens the network of hydrogen bonds which stabilizes the autoinhibitory PH–kinase domain interface. Although we cannot exclude a model in which the interface dissociates prior to PIP_3_ binding, docking of PIP_3_ to the autoinhibited conformation of MbTEC is entirely compatible with molecular dynamics simulations of BTK that have revealed the orientation of the PH domain when bound to membrane-embedded PIP_3_^19^. Given that PIP_3_ is generated, and remains, in the plasma membrane, and that phosphorylated tyrosine motifs are locally constrained in the cytoplasmic tails of activated receptors, this spatially confines MbTEC signaling to the plasma membrane and temporally confines it to the lifetime of receptor activation. PIP_3_-mediated activation of TEC, coincident with the intramolecular engagement of its SH3 domain with its own polyproline motif, promotes its activation by autophosphorylation, an event that has been proposed to be mediated in *trans* by activation loop exchange^7^. Upon attenuation of the PIP_3_ signal, dissociation of its PH domain from the membrane restores TEC autoinhibition, leading to immediate shutdown of the downstream signaling pathway.

Our finding that the PH domain is sufficient to attenuate >90% of kinase activity in the background of a mutation that mimics allosteric activation of the SH3-SH2-kinase module suggests that PIP_3_ can be considered the master regulator of TEC kinase activity. The coincident detection of each signal in space and time ensures the propagation of the signal with high fidelity and almost certainly limits the available substrate pool to those substrates co-localized to the site of TEC activation on the membrane. This is consistent with the membrane localization of one of the best characterized substrates of BTK, phospholipase C γ^57,58^.

It would be remiss not to acknowledge the discrepancy between our structural model for MbTEC and experimental structures of human BTK that have been described by others^6,7^. Published SAXS data^7,24,25^ and cryo-electron microscopy^7^ on human BTK indicate a much more extended structure than we observe for MbTEC, while crystal structures of full-length BTK or synthetic PH-kinase domain fusion constructs either exhibited no electron density for the PH domain^7^ or divergent contacts with the kinase domain^6,7^. As such, the prevailing model for autoinhibition by the PH domain involves an ensemble of different conformational states. However, caution must be exercised in the interpretation of published crystal structures, which may reflect the influence of lattice packing interactions in which enthalpically favorable interactions compensate for the reduction in entropy upon lattice formation. In the case of the solution scattering of BTK, we suspect that the scattering may reflect partial dimerization of BTK at the high concentrations required for SAXS. PH domain-mediated dimerization of BTK via insertion loops specific to BTK (Supplementary Fig. 10A) has been extensively characterized and is supported by crystal structures of the so-called Saraste dimer^16,17^ (Supplementary Fig. 10B). Here, it should be noted that PIP_3_-dependent dimerization of the PH domain of BTK, but not those of TEC or ITK, is required for BTK autophosphorylation and downstream signaling in B cells^6,14,19,59^, indicating that paralog-specific evolutionary adaptations likely created additional layers of control over dimerization, kinase activation, and, ultimately, signaling specificity.

While this study does not address the role of adapter proteins in TEC kinase signaling, it offers new perspectives on their functions. Although our data is insufficient to exclude the possibility that adapter proteins such as Grb2 directly activate TEC kinases^60^ in cells, the high local concentration of the PRR and SH3 domain (intra-TEC) favors a TEC-autonomous model (Fig. 6). Indeed, the *cis*-interaction of the polyproline motif in ITK has been shown to prevent the binding of SH3 domain-containing adapter proteins such as Sam68 and Grb2^39^. In this sense, it is possible that adapter proteins, such as Grb2, govern the subcellular localization of autoinhibited TEC kinases, whose activation is then accomplished in a PI3K-dependent and TEC-intrinsic manner. Such a model is consistent with the presence of a Grb2 ortholog in choanoflagellates^61^ and would provide a coincidence-based mechanism for the discrete subcellular localization of TEC kinases with PI3K prior to their activation. In conclusion, the TEC kinases are exquisitely tuned detectors that function in much the same way that an AND logic gate works in an electronic circuit.

Finally, it is worth noting that the regulation of MbTEC by PIP_3_ is similar to the regulation of the Ser/Thr kinases Akt and PDK1. Like Tec, Akt and PDK1 contain PIP_3_-sensing PH domains which mediate autoinhibition of their respective kinase domains^62,63^. Although the autoinhibitory interfaces of Tec and Akt are structurally different, both interfaces impair activation loop phosphorylation and substrate binding, as well as PIP_3_ binding^62,64,65^. The specific autoinhibitory conformation of Akt has been exploited in the development of allosteric inhibitors, which exhibit significantly improved on-target specificity and have recently been approved for the treatment of cancer^66^. As such, our findings open a new potential therapeutic modality for the development of selective Tec kinase inhibitors. Given the recent success of ATP-competitive BTK inhibitors in treating B cell malignancies^67,68^, there is enormous therapeutic potential.

## Methods

### Structure prediction using AlphaFold 3

A curated list of 2442 MbTEC homologs was generated by filtering UniProt for sequences containing the following domains, as defined by InterPro: PH (IPR001859), Btk_Zn_finger (IPR001562), SH3 (IPR001452), SH2 (IPR000980), and tyrosine kinase (IPR020635). A mass range of 70,000–90,000 Da was included to filter out sequences of excessive length. The sequences were aligned using MAFFT^34,35^ with default settings (BLOSUM62 scoring matrix, gap opening penalty: 1.53, offset value: 0.0) and converted into the A3M format required by AlphaFold. The custom MSA was either fed directly into the database search step of AlphaFold 3^31^ or further curated by taxonomic reweighting or downsampling. For taxonomic reweighting, individual sequences in the MSA were reweighted according to their taxonomic representation such that individual sequences in over-represented taxa were downweighted for the structure inference step. All 2442 sequences were retained in the reweighted MSA.

To reduce bias from over-represented taxa while preserving evolutionary diversity, the alignment was taxonomically downsampled at the *family* level using a custom Python pipeline. Each sequence i was assigned a raw weight

1 $${w}_{i}^{{\rm{raw}}}=\frac{1}{{n}_{{\rm{family}}(i)}}$$

where ${n}_{{\rm{family}}(i)}$ is the total number of sequences from the same taxonomic family as sequence i. The raw weights were normalized with

2 $${w}_{i}=\frac{{w}_{i}^{{\rm{raw}}}}{{\sum }_{j}{w}_{j}^{{\rm{raw}}}}$$

so that all weights sum to 1. Sequences from different families were randomly sampled proportionally to the normalized weights until reaching a target alignment size (1000 sequences). The effective number of sequences (${N}_{{\rm{eff}}}$) in the MSA was calculated from the sampling weights as

3 $${N}_{\mathrm{eff}}=\frac{{\sum }_{i}{w}_{i}}{ma{x}_{i}{w}_{i}}$$

which, for normalized weights, reduces to ${N}_{{\rm{eff}}}=1/{\max }_{i}{w}_{i}$. *N*_eff_ reflects the diversity of the weighted alignment and is commonly used as a proxy for the amount of independent evolutionary information available for co-evolutionary and structure-prediction methods. The procedure and the definition of *N*_eff_ follow those used in previous work on evolutionary-coupling-based structure prediction^51,52^. Calculation of *N*_eff_ before and after taxonomic downsampling revealed a reduction from 719 to 295, representing fewer but more diverse sequences. The custom MSA (2442 sequences, no reweighting or downsampling), reweighted MSA (2442 sequences, reweighted by taxonomic family), or balanced MSA (1000 sequences, downsampled by taxonomic family) was fed into the structure inference pipeline of AlphaFold 3.

For each curated MSA, 100 models were predicted from random seeds. A seed in AlphaFold 3 represents a random starting pose, from which the final model is obtained by diffusion^31^. Model convergence was assessed by calculating the Euclidean distances of residues on the kinase domain to residues on the PH, SH3 and SH2 regulatory domains (Supplementary Fig. 3E), using the formula

4 $${d}_{1,2}\,=\,\sqrt{{({x}_{1}-{x}_{2})}^{2}+\,{({y}_{1}\,-\,{y}_{2})}^{2}+\,{({z}_{1}\,-\,{z}_{2})}^{2}\,}$$

with *x*, *y*, and *z* being the residue Cα coordinates. The agreement with experimental data was assessed by calculation of the root mean square deviation over corresponding main chain Cα atoms for known experimental structures of the SH3-SH2-KD (32K) module. Finally, the models were evaluated by AlphaFold ranking score and the PAE plot for the best scoring model.

### Protein expression and purification

The sequence for *M. brevicollis* MX1 TEC was obtained from MONBRscaffold_8 20,778 to 23,025 (−). MbTEC^FL^ (1–667) and MbTEC^32K^ (229–667) were cloned into the pFastBac Dual vector with an N-terminal GST tag followed by a TEV cleavage site for expression in baculovirus-infected Sf9 insect cells. MbTEC mutants were generated by site-directed mutagenesis.

Cells were centrifuged at 4000 × *g* for 30 min and the pellet lysed in 100 mL lysis buffer (50 mM HEPES pH 7.4, 150 mM NaCl, 1 mM TCEP, 0.25% CHAPS, 2 mM MgCl_2_, 1× protease inhibitor cocktail (Sigma P8849)) per 1 L expression culture. The lysate was centrifuged at 39,000 × *g* for 30 min at 4 °C and the supernatant was incubated with 5 mL glutathione Sepharose beads (Cytiva) for 2 h at 4 °C. The beads were washed three times with Buffer A (50 mM Hepes pH 7.4, 50 mM NaCl, 1 mM TCEP, 0.25% CHAPS), three times with Buffer B (50 mM Hepes pH 7.4, 300 mM NaCl, 1 mM TCEP, 0.25% CHAPS) before being resuspended in Buffer C (50 mM Hepes pH 7.4, 150 mM NaCl, 1 mM TCEP, 0.25% CHAPS). The protein was cleaved off the beads by adding 8.8 nmol TEV protease (purified in-house) and dephosphorylated by adding 10 nmol lambda phosphatase and 13 nmol YopH phosphatase (both purified in-house) overnight at 4 °C. Cleaved MbTEC protein was separated from the beads and diluted to 25 mM NaCl in Buffer Q_A_ (50 mM HEPES, pH 7.4, 1 mM TCEP). The protein was loaded onto a MonoQ anion exchange column (Cytiva). After gradient elution with Buffer Q_B_ (50 mM Hepes pH 7.4, 500 mM NaCl, 1 mM TCEP) from 0 to 50% B in 520 column volumes (CV) to separate differentially phosphorylated protein species, the peak fractions were pooled, concentrated, and injected onto a S200 10/300 GL SEC column (Cytiva) equilibrated in SEC Buffer (20 mM HEPES pH 7.4, 150 mM NaCl, 2% glycerol, 1 mM TCEP). The phosphorylation state of every construct was verified by intact mass spectrometry.

MbTEC^PH^ (1–174) was cloned into the pGST parallel expression vector with an N-terminal GST tag followed by a TEV cleavage site for expression in BL21 STAR bacterial cells. The cells were grown in LB medium containing 100 μg/mL ampicillin at 37 °C, 200 rpm to an OD = 0.7 before induction with 250 μM IPTG and incubation overnight at 20 °C. The cells were centrifuged at 4000 × *g* for 30 min and the pellet lysed in 50 mL lysis buffer (50 mM HEPES pH 7.4, 150 mM NaCl, 1 mM TCEP, 0.25% CHAPS, 2 mM MgCl_2_, 0.2 mg/mL lysozyme, 2 mM benzamidine, 1% glycerol) per 1 L expression culture. The lysate was sonicated, centrifuged at 39,000 × *g* for 30 min at 4 °C and the supernatant was incubated with 5 mL glutathione Sepharose beads (Cytiva) for 2 h at 4 °C. The beads were washed three times with Buffer A (50 mM Hepes pH 7.4, 50 mM NaCl, 1 mM TCEP, 0.25% CHAPS), three times with Buffer B (50 mM Hepes pH 7.4, 300 mM NaCl, 1 mM TCEP, 0.25% CHAPS) before being resuspended in Buffer C (50 mM Hepes pH 7.4, 150 mM NaCl, 1 mM TCEP, 0.25% CHAPS). The protein was cleaved off the beads by adding 8.8 nmol TEV protease (purified in-house) overnight at 4 °C. Cleaved MbTEC^PH^ protein was separated from the beads and diluted to 30 mM NaCl in Buffer S_A_ (50 mM HEPES, pH 7.4, 1 mM TCEP). The protein was loaded onto a CaptoS cation exchange column (Cytiva). After gradient elution with Buffer S_B_ (50 mM HEPES pH 7.4, 500 mM NaCl, 1 mM TCEP) from 0 to 100% B in 50 CV, the peak fractions were pooled, concentrated, and injected onto a S75 10/300 GL SEC column (Cytiva) equilibrated in SEC Buffer (20 mM HEPES pH 7.4, 150 mM NaCl, 2% Glycerol, 1 mM TCEP).

### Thermal stability assays

Differential scanning fluorimetry measurements were performed in SEC buffer with 5x SYPRO Orange (Life Technologies) using a BioRad iQ5 Multicolor Real-Time PCR detection system. For each sample, the protein was diluted to 0.1 mg/mL either in the presence or absence of 1 mM ADP and 2 mM MgCl_2_. The samples were heated from 25 °C to 90 °C in increments of 0.5 °C. The melting temperature was determined from the second derivative of the melting curve and averaged from three independent measurements.

### Mass photometry

Microscopy coverslips were cleaned by sequential sonication in water, isopropanol and water before drying under an air stream. Prior to measurements, CultureWell gaskets (Merck) were mounted onto the coverslip. Protein samples were clarified by centrifugation (5 min, 21,000 × *g*, 4 °C diluted to 0.5 μM in SEC buffer. To find focus, 19 μL of buffer were added into a chamber, the focal position was identified and secured with an autofocus system. Then, 1 μL of 0.5 μM protein sample was added, mixed and movies of 60 s duration were recorded. All measurements were performed using a Two^MP^ mass photometer (Refeyn) and analyzed using the software Discover MP (Refeyn). Contrast-to-mass calibration was performed before every experiment.

### SEC-MALS

The oligomeric state of the PH domain construct was assessed by SEC-MALS. Fifty μL of PH domain (3 mg/mL) was injected onto a Superdex 75 10/300 column (Cytiva) connected to a 1260 Infinity HPLC (Agilent Technologies) equilibrated in 50 mM HEPES pH 7.4, 150 mM NaCl, 1 mM TCEP. Light scattering was detected with a MiniDawn Treos (Wyatt) and the refractive index was measured with a Shodex RI-101 (Shodex) detector.

### Fluorescence anisotropy

For fluorescence anisotropy measurements of affinity, the MbTEC^PH^ was labeled with maleimide-Atto488 (ATTO-TEC) as previously reported^64^. Unreacted dye was removed by gel filtration and labeling was confirmed by mass spectrometry (Supplementary Fig. 1N). MbTEC^32K^ constructs were serially diluted in 20 mM HEPES pH 7.4, 150 mM NaCl, 2% glycerol and 1 mM TCEP containing 200 nM of the labeled PH domain. Measurements were performed with a FS5 Spectrofluorometer (Edinburgh Instruments) equipped with a QS High Performance cuvette (Hellma). For each concentration, 10 technical replicates were measured at a controlled temperature of 21 °C. Excitation and emission wavelengths were set to 501 nm and 523 nm, with a bandwidth of 5 nm for both and an exposure time of 1 s. Affinities were determined by fitting a one-site binding model in GraphPad Prism.

### Radiometric kinase assay

All radiometric kinase assays were performed using radiolabeled [γ^32^ P] ATP (Hartman Analytic) at room temperature (RT) in the same buffer (20 mM HEPES pH 7.4, 150 mM NaCl, 2% glycerol, 1 mM TCEP, 1 mM ATP and 2 mM MgCl_2_ spiked with 1:50 [γ^32^ P] ATP) and a final protein concentration of 0.5–2 μM. After clarifying the dephosphorylated protein sample, it was incubated for the indicated duration. At each time point, a 10 μL sample was collected and the reaction was quenched by adding 2.5 μL of 1 M EDTA and 3 μL 5× SDS Loading Dye. The samples were separated by SDS-PAGE to remove non-incorporated ATP. The gel was washed 3×5 min with water, dried and exposed to a phosphor screen overnight. The screen was imaged with an Amersham Typhoon imager and the radioactive signal was quantified with ImageJ. The fraction of phosphorylated MbTEC protein was calculated from an internal standard, assuming the incorporation of one phosphate per molecule MbTEC (verified by intact mass spectrometry).

For kinase assays containing nanodiscs doped with either PIP_2_ or PIP_3_, MbTEC was incubated with nanodiscs in a fourfold excess of protein (assuming a maximum binding stoichiometry of 4:1 MbTEC:nanodisc) for 1 h at RT prior to the addition of ATP and MgCl_2_. The final protein concentration was 1 μM.

### ADP-Glo kinase assay

Kinase assays were performed using the ADP-Glo kit (Promega) according to the manufacturer’s instructions. Kinase reactions contained 500 nM purified kinase, 50 μM SRC substrate (SignalChem #S30-58), and 1 mM ATP in 20 mM Tris pH 7.5, 100 mM NaCl, 1 mM TCEP, 2 mM MgCl_2_, and 0.05 mg/ml BSA. Reactions were incubated at RT for the specified time and luminescence was measured using a TECAN Infinite F500 plate reader.

### SAXS

SAXS data for MbTEC^FL^ and MbTEC^32K^ were collected on BM29 at the ESRF, Grenoble, France using an in-line SEC-SAXS setup. Proteins were applied to a Agilent Bio SEC 300 column equilibrated in 20 mM Tris, pH 7.4, 150 mM NaCl, 1 mM TCEP and 1% (v/v) glycerol and images were acquired every second for the duration of the size exclusion run. Buffer subtraction was performed by averaging 50 frames either side of the peak. All subsequent data processing steps were performed using the ATSAS data analysis software 3.9.1. The program DATGNOM^69^ was used to generate the pair distribution function [P(r)] for each isoform and to determine *D*_max_ and *R*_g_ from the scattering curves [*I*(*q*) vs. *q*] in an automatic, unbiased manner. For ab initio calculation of the molecular envelopes, iterative rounds of dummy atom modeling were followed by alignment of the resulting models, trimming of outliers, and refinement of the core electron density, followed by superposition of the respective models for MbTEC^FL^ and MbTEC^32K^.

### Mass spectrometry

Intact protein samples were diluted in H_2_O and up to 100 ng protein were loaded on an XBridge Protein BEH C4 column (2.5 µm particle size, dimensions 2.1 mm × 150 mm; Waters) using a Dionex Ultimate 3000 HPLC system (Thermo Fisher Scientific) with a working temperature of 50 °C, 0.1% formic acid (FA) as solvent A, 80% acetonitrile, 0.08% FA as solvent B. Proteins were separated with a 6 min step gradient from 10 to 80% solvent B at a flow rate of 300 μL/min and analyzed on a Synapt G2-Si coupled via a ZSpray ESI source (Waters). Data were recorded with MassLynx V 4.1 (Waters) and analyzed using the MaxEnt 1 process to reconstruct the uncharged average protein mass.

For tandem mass spectrometry analysis of samples, 2 μg protein were precipitated overnight at −20 °C by adding ice-cold acetone to a concentration of 80%. The proteins were pelleted by centrifugation at 16,000 × *g* for 10 min at 18 °C. The pellets were dissolved in 2% sodium deoxycholate (SDC) in 50 mM ammonium bicarbonate (ABC). Proteins were reduced by adding 250 mM dithiothreitol (DTT) to final concentration of 10 mM for 30 min at 50 °C and alkylated by adding 500 mM iodoacetamide (IAA) to final concentration of 20 mM and incubating for 30 min at RT in the dark. The remaining IAA was quenched for 10 min by adding 5 mM DTT. The samples were diluted to 1% SDC with 20 µL 50 mM ABC. Proteins were digested by adding 60 ng trypsin (Trypsin Gold, Promega; 100 ng/μL solution in 1 mM HCl) and incubated at 37 °C overnight. The digest was stopped by the addition of 10% trifluoroacetic acid (TFA) to reach a final concentration of 2%. SDC was pelleted by centrifugation at 18,500 × *g* for 10 min. The resulting supernatant was desalted using C18 Stagetips^70^.

Liquid chromatography tandem mass spectrometry analysis was performed on a Vanquish Neo UHPLC system (Thermo Scientific) coupled to an Orbitrap Exploris 480 mass spectrometer (Thermo Scientific). The system was equipped with a Nanospray Flex ion source (Thermo Scientific), coated emitter tips (PepSep, MSWil), and a Butterfly Portfolio Heater (Phoenix S&T).

Peptides were loaded onto a trap column (Acclaim PepMap 100 C18 HPLC Column, 20 mm × 0.1 mm, 5 μm particle size, Thermo Scientific) using 0.1% TFA as mobile phase, and separated on an analytical column (Acclaim PepMap 100 C18 HPLC Column, 50 cm × 75 µm, 2 μm particle size, Thermo Scientific), applying a linear gradient starting with a mobile phase of 98% solvent A (0.1% FA) and 2% solvent B (80% acetonitrile, 0.08% FA), increasing to 35% solvent B over 60 min at a flow rate of 230 nl/min. The analytical column was heated to 30 °C.

The mass spectrometer was operated in data-dependent acquisition (DDA) mode, with 2 s MS1 cycle time. Survey scans were acquired from 375 to 2000 *m*/*z* with lock mass enabled, normalized AGC target of 300%, resolution of 120,000. The most intense precursor ions (charge states +2 to +6) were selected for fragmentation using an isolation window of 1.4 *m*/*z*. Selected ions were analyzed with a maximum fill time of 200 ms, normalized AGC target of 200%, and resolution of 30,000 after HCD fragmentation with normalized collision energy of 30%. Monoisotopic precursor selection was set to “peptide” mode with relax restrictions on, the intensity threshold to 2.5 E4, and selected precursors were dynamically excluded for 20 s with isotope exclusion enabled.

MS raw data were analyzed with FragPipe (22.0), using MSFragger (4.1)^71^, IonQuant (1.10.27)^72^, and Philosopher (5.1.1)^73^. The default FragPipe workflow was used, turning on MS1 Quant omitting normalization and MBR. Cleavage specificity was set to Trypsin/P, with two missed cleavages allowed. The ion, peptide and protein false discovery rate was set to 1%. Carbamidomethyl was used as fixed cysteine modification; methionine oxidation, protein N-terminal acetylation, and phosphorylation of serine, threonine and tyrosine were specified as variable modifications. MS2 spectra were searched against the *S. frugiperda* entries from Uniprot (Taxon ID: 7108, release 2024.01), concatenated with a database of 379 common laboratory contaminants (release 2023.03, https://github.com/maxperutzlabs-ms/perutz-ms-contaminants), and the entries for the MbTEC constructs.

Computational analysis was performed using Python and the Python library MsReport (0.0.26)^74^. Protein intensities were log2 transformed; phosphorylation sites with a localization probability <80% were filtered out. The Python library XlsxReport (0.1.1)^75^ was used to create formatted Excel files summarizing the results of the proteomics experiments. The proteomics data have been deposited to the ProteomeXchange Consortium via the PRIDE partner repository^76^ with the dataset identifier PXD064005.

### HDX-MS

HDX reactions comparing MbTEC^FL^ to either MbTEC^FL^ Y579D R581E, MbTEC^32K^ construct or MbTEC^PH^ were carried out in a 20 µl reaction volume containing 15 pmol (MbTEC^FL^ vs MbTEC^32K^ and MbTEC^FL^ vs MbTEC^PH^) or 20 pmol (MbTEC^FL^ vs MbTEC^FL^ Y579D R581E) for each respective condition. For experiments with MbTEC^32K^ and MbTEC^PH^, reactions were initiated by the addition of 16.6 µL of D_2_O buffer (20 mM HEPES pH 7.5, 150 mM NaCl, 1 mM TCEP, 95.1% D2O (V/V)) to 3 µL of protein and 0.4 μL of MgCl_2_ + ADP (final D_2_O concentration of 78.9%, final MgCl_2_ concentration of 2 mM, final ADP concentration of 1 mM). Reactions proceeded for 3 s at 0°C, and 30 s, 300 s and 3000 s at 20 °C before being quenched with ice-cold acidic quench buffer, resulting in a final concentration of 0.6 M guanidine HCl and 0.9% FA post quench. The exchange reactions for MbTEC^FL^ vs MbTEC^FL^ Y579D R581E were initiated by the addition of 13.5 µl of D_2_O buffer (20 mM HEPES pH 7.4, 150 mM NaCl, 1 mM TCEP 95.1% D_2_O (V/V)) to 2.5 µL of protein and 4.0 µL of MgCl_2_ + ADP (final D_2_O concentration of 64.2%, final MgCl_2_ concentration of 2 mM, final ADP concentration of 1 mM). Reactions proceeded for 3 s at 0 °C, and 3 s, 30 s, 300 s, and 3000 s at 20 °C before being quenched with ice cold acidic quench buffer, resulting in a final concentration of 0.6 M guanidine HCl and 0.9% FA post-quench. All conditions and timepoints were created and run in independent triplicate. Samples were flash frozen immediately after quenching and stored at −80 °C until injected onto the ultra-performance liquid chromatography (UPLC) system for proteolytic cleavage, peptide separation, and injection onto a QTOF for mass analysis, described below.

Protein samples were rapidly thawed and injected onto an integrated fluidics system containing a HDx-3 PAL liquid handling robot and climate-controlled (2 °C) chromatography system (LEAP Technologies), a Waters Acquity UPLC I-Class Series System, as well as an Impact HD QTOF Mass spectrometer (MbTec^FL^v MbTec^32K^ or MbTec^FL^ v MbTec^PH^) (Bruker) or an Impact II QTOF Mass spectrometer (MbTec^FL^ v MbTec^FL^ Y579D R581E). The full details of the automated LC system were previously described in ref. ^77^. The samples were run over an immobilized pepsin column (Affipro: AP-PC-001) at 200 µL/min for 4 min at 2 °C. The resulting peptides were collected and desalted on a C18 trap column (Acquity UPLC BEH C18 1.7 µm column (2.1 × 5 mm); Waters 186004629). The trap was subsequently eluted in line with an ACQUITY 300 Å, 1.7 μm particle, 100 × 2.1 mm BEH C18 UPLC column (Waters), using a gradient of 3–10% B (Buffer A 0.1% FA; Buffer B 100% acetonitrile) over 1.5 min, followed by a gradient of 10–25% B over 4.5 min, followed by a gradient of 25–35% B over 5 minutes, finally after 1 min at 35% B a gradient of 35-80% B over 1 min was used. Mass spectrometry experiments acquired over a mass range from 150 to 2200 *m*/*z* using an electrospray ionization source operated at a temperature of 200 °C and a spray voltage of 4.5 kV.

Peptides were identified from the non-deuterated samples of full length MbTEC using data-dependent acquisition following tandem MS/MS experiments (0.5 s precursor scan from 150 to 2000 *m*/*z*; 12 0.25 s fragment scans from 150 to 2000 *m*/*z*). MS/MS datasets were analyzed using FragPipe v18.0 (MbTec^FL^v MbTec^32K^ or MbTec^FL^ v MbTec^PH^) or FragPipe v23.1 (MbTec^FL^ v MbTec^FL^ Y579D R581E) peptide identification was carried out by using a false discovery-based approach using a database of purified proteins and known contaminants^71,73,78^. MSFragger was utilized, and the precursor mass tolerance error was set to −20 to 20 ppm. The fragment mass tolerance was set at 20 ppm. Protein digestion was set as nonspecific, searching between lengths of 4 and 50 aa, with a mass range of 400–5000 Da.

HD-Examiner Software (Sierra Analytics) was used to automatically calculate the level of deuterium incorporation into each peptide. All peptides were manually inspected for correct charge state, correct retention time, appropriate selection of isotopic distribution, etc. Deuteration levels were calculated using the centroid of the experimental isotope clusters. Results are presented as relative levels of deuterium incorporation and the only control for back exchange was the level of deuterium present in the buffer (78.9% or 64.2%). Differences in exchange in a peptide were considered significant if they met all three of the following criteria: ≥5% change in exchange, ≥0.4 Da difference in exchange, and a *p* value <0.01 using a two-tailed Student’s *t* test. Samples were only compared within a single experiment and were never compared to experiments completed at a different time with a different final D_2_O level. The data analysis statistics for all HDX-MS experiments are in the Source data file according to published guidelines^79^. The mass spectrometry proteomics data have been deposited to the ProteomeXchange Consortium via the PRIDE partner repository^76^ with the dataset identifier PXD069610.

### Fluorescence microscopy

NIH/3T3 fibroblasts (ATCC Cat# CRL-6442, RRID:CVCL_0594) were cultured according to the manufacturer’s instructions. Briefly, cells were cultured in Dulbecco’s Modified Eagle Medium (DMEM; Gibco) supplemented with 10% fetal bovine serum (FBS; Sigma-Aldrich) and Pen/Strep (Fisher Scientific) and passaged every 2 days. For transient transfection, they were seeded sub-confluently and co-transfected with two plasmids encoding mCherry-PH domain or eGFP-FL WT on the following morning with Lipofectamine 300 according to the manufacturer’s instructions. After 7 h of recovery, the media was replaced to DMEM without FBS for overnight starvation until microscopy imaging. During imaging in FluoroBrite media (Gibco), the media was spiked with 100 ng/mL of PDGFαβ (Gibco).

Cells were imaged using a Zeiss LSM-980 confocal microscope with the Airyscan 2 module. Acquisition settings were chosen to satisfy the necessary 2× Nyquist sampling requirement for Airyscan imaging. Three-dimensional stacks were acquired over 40 time points with 30-s intervals. The integrated Airyscan 2 multiplexing functionality was employed to achieve faster imaging speeds. Maximum intensity projections of the *Z*-stacks were subsequently generated for visualization.

Representative regions of interest, where strong responses were observed, were first extracted from raw files. Analysis was then performed within these ROIs. Raw images were first denoised using a 5 × 5 median filter, followed by background subtraction via white-tophat transform with a rolling ball-disk (radius 7 px). As MbTEC fluorescence was observed to accumulate in tube-like structures, a Frangi vesselness filter (**α**: 1.0, **γ**: 5.0, **σ**: 6, 8, 10, 12) was applied to both channels to pronounce any filamentous structures.

The frangi filter is a multi-scale, Hessian based ridge-detection algorithm, highlighting tubular structures, while suppressing “blobs” or plate-like features. Three frangi-filtering parameters were specified for this analysis:

- **α:** (“plate sensitivity”) specifies how strongly plate-like structures are weighed down.
- **γ:** (“structuredness”) governs overall noise tolerance, dampening out low contrast regions.
- **σ**: specifies the scale at which the gaussian filter during vessel detection is applied. Selecting a range of sigmas leads to the filter running across multiple scales, while the strongest response across all sigma values is captured.

The two frangi-filtered images were then averaged—resulting in a combined tubeness-response image for both channels. Segmentation was then performed by applying a global Otsu threshold scaled by a factor 0.5, and small objects were filtered ( < 200 px^2^). From these binary masks, features were extracted from the unprocessed image in both channels (area, mean intensity, median intensity). To account for photobleaching during image acquisition, the overall decrease in fluorescence was extracted from the raw files. Extracted intensities from the masked regions were then fitted to the obtained bleaching-curve and normalized to the first timepoint.

### Preparation of PIP_3_-containing nanodiscs

MSP1D1 was purified as previously reported with additional anion exchange chromatography and gel filtration steps. Briefly, the MSP1D1 sequence was derived from a plasmid obtained from AddGene (ID: 173482) and cloned into pET28a vector with a N-terminal His-tag. The plasmid was transformed into *E. coli* BL21 Star. The cells were grown in LB supplemented with 100 µg/mL ampicillin at 37 °C and 180 rpm to an OD_600_ of 0.7. Expression was induced with 0.2 mM IPTG and the culture was incubated overnight at 18 °C and 180 rpm.

The cells were harvested by centrifugation at 4000 rpm for 30 min and frozen in liquid nitrogen. The pellet was resuspended in lysis buffer (50 mM Tris pH 8.0, 400 mM NaCl, 5% glycerol, 1 mM MgCl_2_, 0.25% CHAPS, 5 mM imidazole, 2 mM DTT) supplemented with 250 U of Denarase (c-LEcta) and sonicated three times at 50% for 3 min with 1 sec on and off pulse. The lysate was cleared by centrifugation at 18,000 rpm for 30 min and loaded onto a Ni^2+^-NTA column (HisTrap 5 mL FF, Cytiva) pre-equilibrated in 50 mM Tris pH 8, 400 mM NaCl, 5% glycerol, 5 mM imidazole, 2 mM DTT. The column was washed with 10 CV of 50 mM Tris pH 8, 400 mM NaCl, 5% glycerol, 20 mM imidazole, 2 mM DTT and followed by a step-gradient to 50 mM Tris pH 8, 400 mM NaCl, 5% glycerol, 500 mM imidazole, 2 mM DTT for elution of MSP1D1. The sample was dialyzed overnight against 50 mM Tris pH 8.0, 100 mM NaCl, 5% glycerol, 1 mM DTT, while simultaneously cleaving-off the His-tag using His-TEV protease (in-house). The dialyzed sample was loaded over a Ni^2+^-NTA column (HisTrap 5 mL FF, Cytiva) for removal of the cleaved tag and protease. The flow-through was subjected to anion exchange chromatography (HiTrap Q 1 mL HP, Cytiva), and subsequent gel filtration using a 16/600 Superdex 75 pg column pre-equilibrated in 50 mM Tris pH 8, 100 mM NaCl, 5% glycerol, 1 mM TCEP. The protein was concentrated, snap-frozen in liquid nitrogen, and stored at −70 °C until further use.

Nanodiscs were prepared as previously reported^80^ with lipids purchased from Avanti Polar Lipids. Briefly, chloroform stocks of cholesterol (5 mg/mL), DOPC (5 mg/mL), DOPS (10 mg/mL), and DOPE (5 mg/mL) were prepared, whereas PIP_3_ was solubilized in a chloroform:methanol:water (1:2:0.8) mixture. Lipids were combined in a borosilicate glass tube (Duran) at the following molar ratio: 20% cholesterol, 15% DOPS, 20% DOPC, 35% DOPE, and 10% PIP_3_. The lipids were dried under a nitrogen stream, incubated under vacuum for 2 h and rehydrated in buffer A (50 mM Tris pH 8, 100 mM NaCl, 1 mM TCEP). The mixture was freeze-thawed 5× with vortexing and sonication steps in between (Bandelin Sonorex Digiplus, 90% for 5 min at RT). The sample was supplemented with 0.5% dodecyl maltoside (DDM) in buffer A and incubated at RT for 1 h. Next, Buffer A and MSP1D1 were added to obtain a MSP1D1:lipid molar ratio of 1:60 with a final concentration of 0.1% DDM. The mixture was incubated on ice for 1 h, followed by gentle rotation overnight at 4 °C on BioBeads SM-2 Absorbents (BioRad #1523920) for removal of the detergent. The nanodiscs were purified on a Superose 6 Increase 3.2/100 column and stored at 4 °C until further use.

## Supplementary information

Supplementary information

Source-Data

## Data Availability

All proteomics data have been deposited to the ProteomeXchange Consortium via the PRIDE partner repository^76^ with the dataset identifiers PXD064005 and PXD069610. Source data are provided with this paper.
