# Supplementary material for: Mechanism of activation of an ancestral TEC kinase by PIP3
Source: Npj Struct Biol. 2026 Oct 1;1(1):1. doi: 10.1038/s44511-026-00001-z (PMC13648279; doi:10.1038/s44511-026-00001-z)
Supplement: Supplementary file 1 [file 44511_2026_1_MOESM1_ESM.pdf]

# Supplementary Data

## Mechanism of activation of an ancestral TEC kinase by $PIP_3$

Eva Krötenheerdt[1,2], Lucas Piëch[1,2], Freia von Raußendorf[1], Ronja Reinhardt[1], Ranjana Nataraj[1,2], Nicholas Wedige[3], Hunter G. Nyvall[4], Emma E. Walsh[4], John E. Burke[4,5,6] and Thomas A. Leonard[1,*]

[1]Max Perutz Labs, Medical University of Vienna, Dr. Bohr-Gasse 9, 1030, Vienna, Austria

[2]Vienna BioCenter PhD Program, a Doctoral School of the University of Vienna and the Medical University of Vienna, A-1030 Vienna, Austria

[3]BioOptics Light Microscopy Facility, Max Perutz Labs, Dr. Bohr-Gasse 9, 1030 Vienna, Austria

[4]Department of Biochemistry and Microbiology, University of Victoria, Victoria, BC, Canada V8W 2Y2

[5]Department of Biochemistry and Molecular Biology, University of British Columbia, Vancouver, British Columbia V6T 1Z3, Canada

[6]University of Victoria Genome BC Proteomics Centre, Victoria, BC, Canada.

[*]Correspondence: thomas.leonard@meduniwien.ac.at

Contains: Supplementary Figures 1-10

# Supplementary Figures

## Supplementary Figure 1. Sequence alignment of hydrophobic stack residues.

**A**

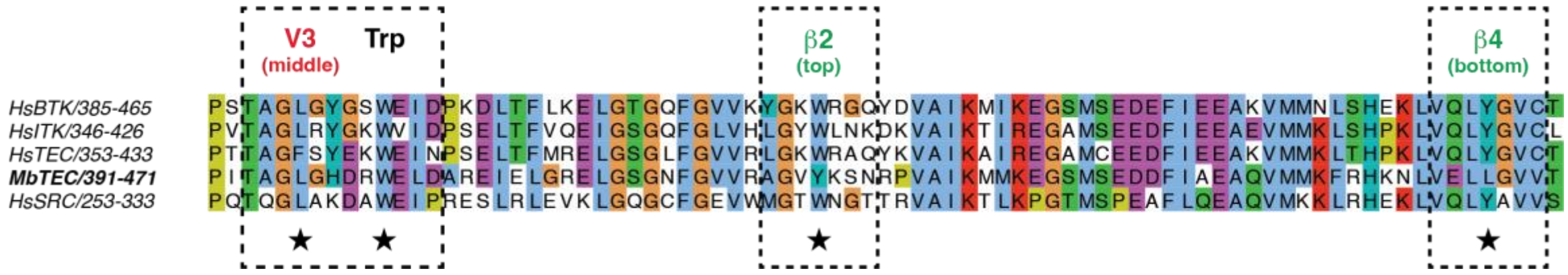


A. Sequence alignment of human Tec paralogs and MbTec covering residues of the hydrophobic stack depicted in Figure 1B-D.

# Supplementary Figure 2. Mass spectrometry of constructs employed in this study.

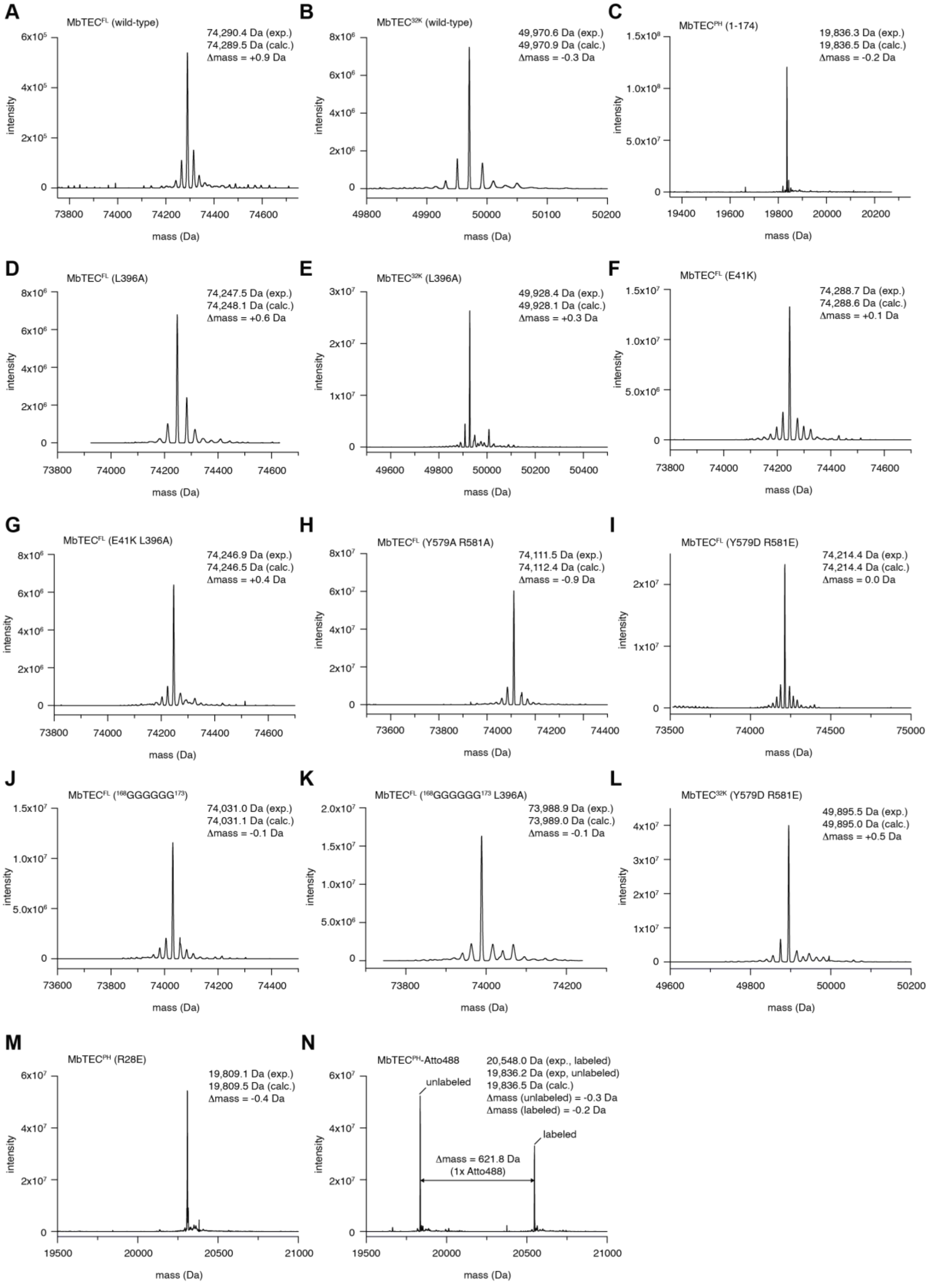

A. Deconvoluted mass spectrum of MbTEC$^{FL}$.

B. Deconvoluted mass spectrum of MbTEC$^{32K}$.

C. Deconvoluted mass spectrum of MbTEC$^{PH}$.

D. Deconvoluted mass spectrum of MbTEC$^{FL}$ L396A.

E. Deconvoluted mass spectrum of MbTEC$^{32K}$ L396A.

F. Deconvoluted mass spectrum of MbTEC$^{FL}$ E41K.

G. Deconvoluted mass spectrum of MbTEC$^{FL}$ E41K L396A.

H. Deconvoluted mass spectrum of MbTEC$^{FL}$ Y579A R581A.

I. Deconvoluted mass spectrum of MbTEC$^{FL}$ Y579D R581E.

J. Deconvoluted mass spectrum of MbTEC$^{FL}$ $^{168}$GGGGGG$^{173}$.

K. Deconvoluted mass spectrum of MbTEC$^{FL}$ $^{168}$GGGGGG$^{173}$ L396A.

L. Deconvoluted mass spectrum of MbTEC$^{32K}$ Y579D R581E.

M. Deconvoluted mass spectrum of MbTEC$^{PH}$ R28E.

N. Deconvoluted mass spectrum of MbTEC$^{PH}$-Atto488.

## Supplementary Figure 3. Biophysical characterization of constructs employed in this study.

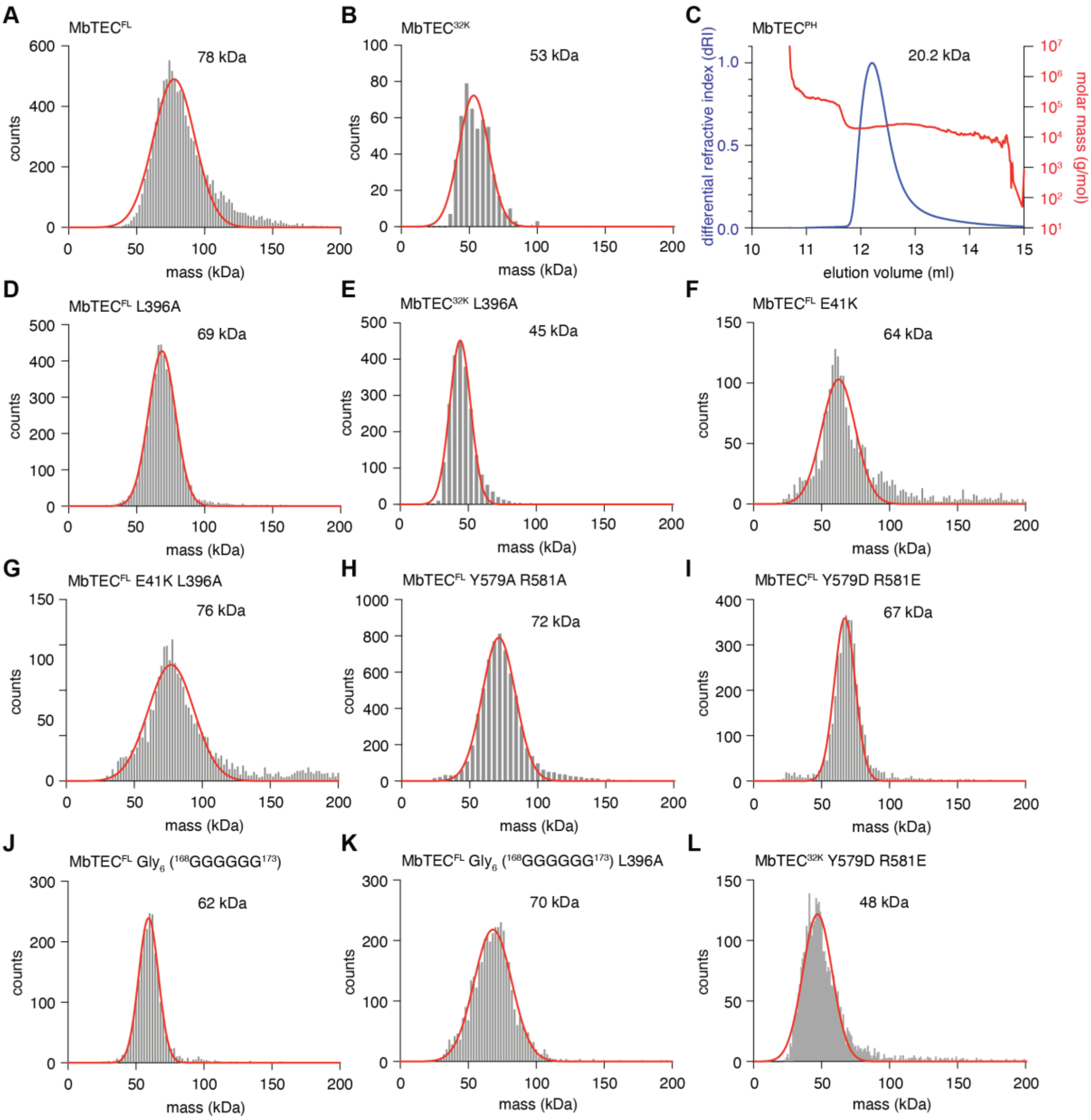


A. Mass photometry of $\text{MbTEC}^{\text{FL}}$.

B. Mass photometry of $\text{MbTEC}^{\text{32K}}$.

C. SEC-MALS analysis of $\text{MbTEC}^{\text{PH}}$.

D. Mass photometry of $\text{MbTEC}^{\text{FL}}$ L396A.

E. Mass photometry of $\text{MbTEC}^{\text{32K}}$ L396A.

F. Mass photometry of $\text{MbTEC}^{\text{FL}}$ E41K.

G. Mass photometry of MbTEC$^{FL}$ E41K L396A.

H. Mass photometry of MbTEC$^{FL}$ Y579A R581A.

I. Mass photometry of MbTEC$^{FL}$ Y579D R581E.

J. Mass photometry of MbTEC$^{FL}$ Gly$_6$ ($^{168}$GGGGGG$^{173}$).

K. Mass photometry of MbTEC$^{FL}$ Gly$_6$ ($^{168}$GGGGGG$^{173}$) L396A.

L. Mass photometry of MbTEC$^{32K}$ Y579D R581E.

## Supplementary Figure 4. *M. brevicollis* TEC adopts a conserved autoinhibited conformation.

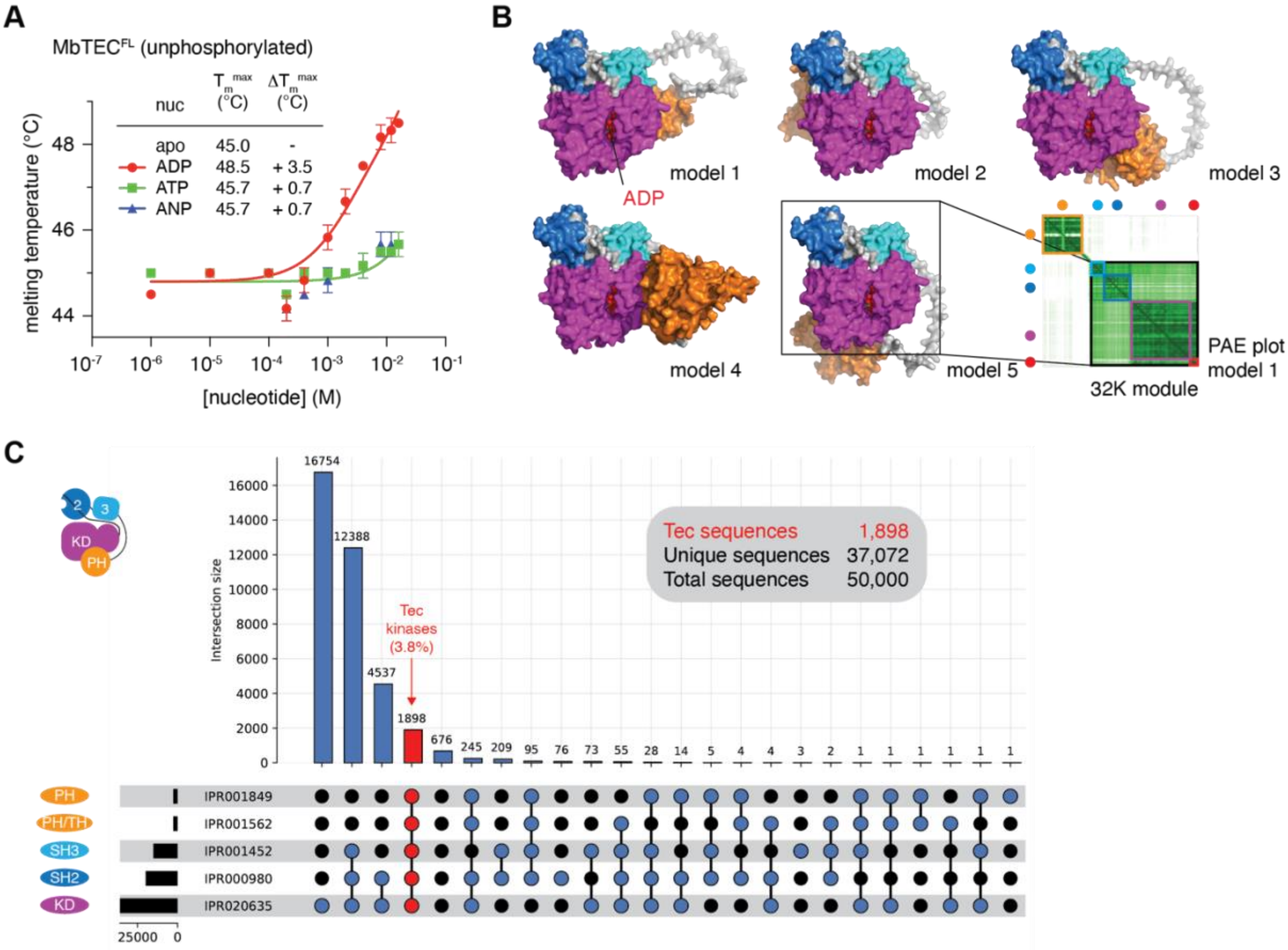


A. Thermal stability measurements of MbTec as a function of nucleotide concentration. ADP (red), ATP (green), AMPPNP (ANP; blue).

B. Five independent AlphaFold 3 predictions of the structure of human Btk. Bottom right: pair alignment error (PAE) plot for model 1.

C. UpSet plot depicting the 37,072 unique sequences curated by AlphaFold3 in its MSA. Partial sequences were mapped back to their parental full-length sequences, which were then binned according to the presence of absence of InterPro domains corresponding to the PH, TH, SH3, SH2 and kinase domains of Tec. Red: 1,898 full-length Tec sequences contain all five domains.

## Supplementary Figure 5. *M. brevicollis* TEC adopts a conserved autoinhibited conformation.

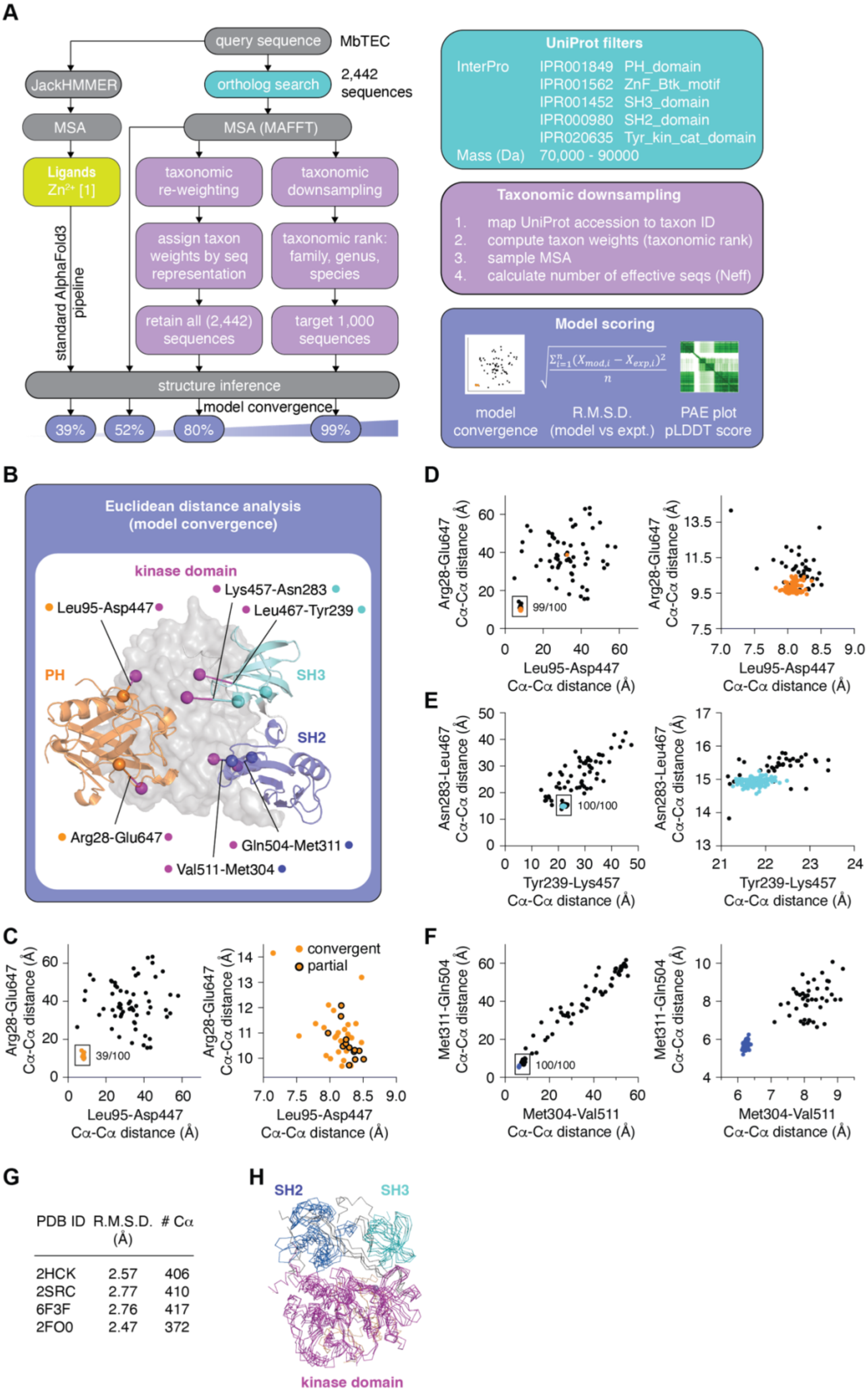

A. Pipeline for custom MSA building, curation, and taxonomic reweighting or downsampling. The custom MSA is then fed into the structure inference step of AlphaFold 3 along with ligands (in the case of MbTEC, a single zinc ion). We scored the output models using three metrics: Euclidean distance plots (convergence on domain arrangements), root mean square deviation (R.M.S.D.) of the model from experimental structures, the PAE plot (pair-aligned residue model confidence). Improvement of custom AlphaFold 3 structure prediction pipelines on model convergence compared to the standard configuration of AlphaFold 3 implemented on the publicly accessible AlphaFold 3 server.

B. Schematic of model evaluation by Euclidean distance measurements for MbTEC. The distance between fixed residue pairs on the kinase domain and each other regulatory domain (PH, SH3, SH2) served as a proxy for the relative domain arrangements in each model. The residue pairs are indicated with colored spheres joined by lines. The color of the sphere indicates the domain to which the residue belongs.

C. Outcome of 100 independent predictions using the AlphaFold 3 server. Input: MbTEC sequence and a single zinc ion. 39/100 models (orange circles) converged on the same arrangement of the PH and kinase domains, of which 26 also converged on the same arrangement of the SH3, SH2 and kinase domains (convergent models). The remaining 13 models (partial convergence) exhibited divergent positions for the SH3 and SH2 domains with respect to the PH and kinase domains (orange circles, black outline).

D. PH-kinase domain Euclidean distance analysis of 100 independent AlphaFold 3 models using the standard pipeline (black circles) compared to our custom pipeline (orange circles).

E. SH3-kinase domain Euclidean distance analysis of 100 independent AlphaFold 3 models using the standard pipeline (black circles) compared to our custom pipeline (cyan circles).

F. SH2-kinase domain Euclidean distance analysis of 100 independent AlphaFold 3 models using the standard pipeline (black circles) compared to our custom pipeline (blue circles).

G. Table of PDB entries for experimentally determined structures of the 32K module of TEC kinases and their corresponding R.M.S.D. from the highest ranked AlphaFold 3 model obtained (model 18).

H. Superposition of the MbTEC32K model with the corresponding structures of Src (2SRC, 6F3F), Hck (2HCK), and Abl (2FO0).

## Supplementary Figure 6. MbTEC adopts a compact conformation in solution.

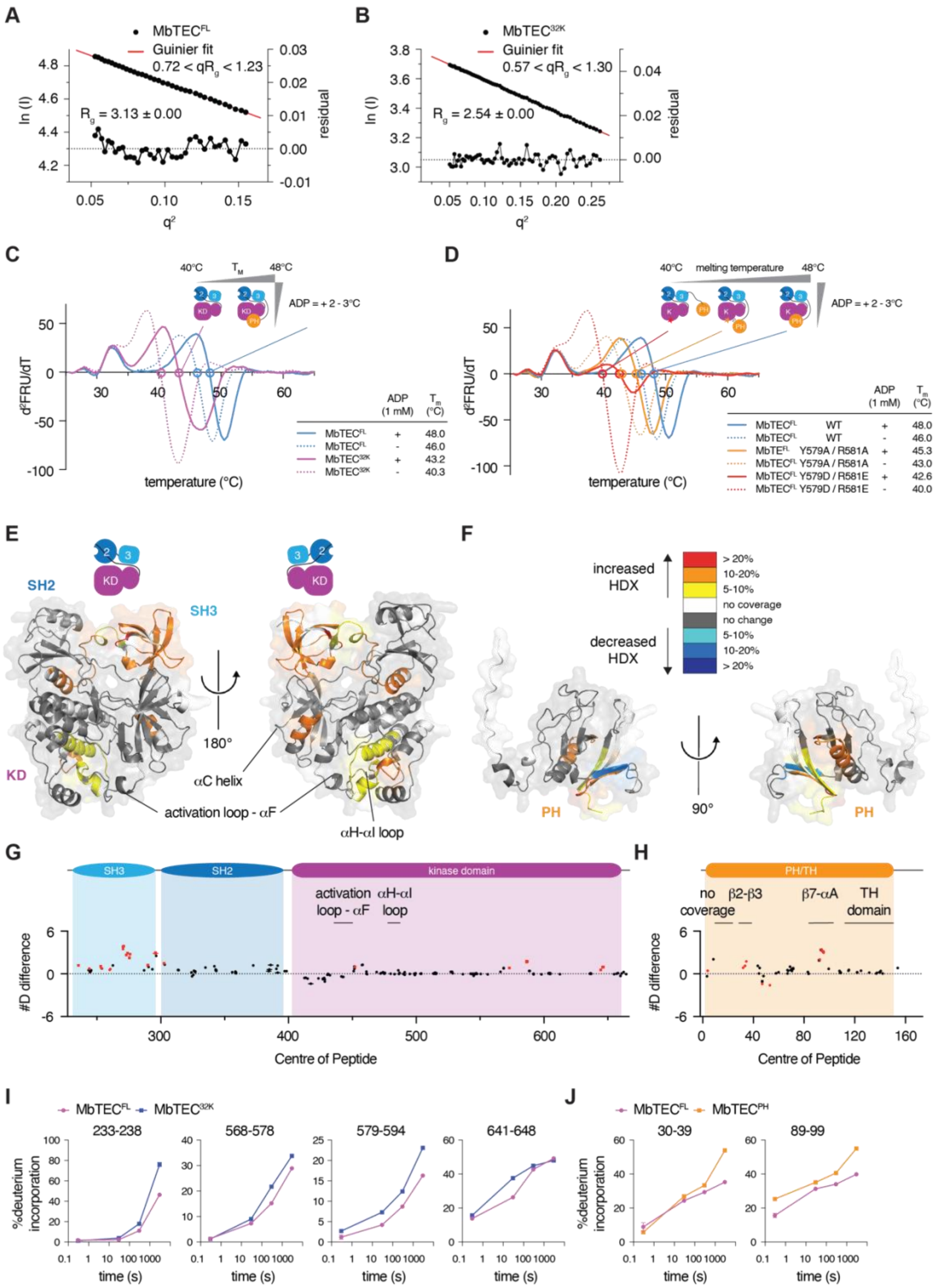


A. Guinier fit of the low-angle scattering regime for MbTEC^FL.

B. Guinier fit of the low-angle scattering regime for MbTEC^32K.

C. Second derivatives of the melting curves for $MbTEC^{FL}$ (blue), $MbTEC^{32K}$ (magenta). Solid lines: + 1mM ADP; dotted lines: -ADP. Inset: table of corresponding MbTEC melting temperatures in the presence and absence of 1 mM ADP and 2 mM $MgCl_2$.

D. Second derivatives of the melting curves for $MbTEC^{FL}$ (blue), $MbTEC^{FL}$ Y579A R581A (orange) and $MbTEC^{FL}$ Y579D R581E (red). Solid lines: + 1mM ADP; dotted lines: -ADP. Inset: table of corresponding MbTEC melting temperatures in the presence and absence of 1 mM ADP and 2 mM $MgCl_2$.

E. Pairwise HDX-MS analysis of $MbTEC^{FL}$ compared to $MbTEC^{32K}$. Significant differences in HDX in $MbTEC^{32K}$ are mapped onto the AlphaFold3 model of $MbTEC^{32K}$ and color coded according to the legend.

F. Pairwise HDX-MS analysis of $MbTEC^{FL}$ compared to $MbTEC^{PH}$. Significant differences in HDX in $MbTEC^{PH}$ are mapped onto the AlphaFold3 model of $MbTEC^{PH}$ and color coded according to the legend.

G. Sum of #D difference in deuterium incorporation for $MbTEC^{32K}$ over the entire time course. Each point represents and individual peptide with significant changes shown in red (greater than 0.40 Da, 5% difference and two tailed t-test $p<0.01$ at any timepoint, n=3). Domain architecture is annotated above.

H. Sum of #D difference in deuterium incorporation for $MbTEC^{PH}$ over the entire time course. Each point represents an individual peptide with significant changes shown in red (greater than 0.40 Da, 5% difference and two tailed t-test $p<0.01$ at any timepoint, n=3). Domain architecture is annotated above. The full deuterium exchange information for all peptides is included in the source data.

I. Deuterium incorporation plots for selected significant peptides shown in panel G.

J. Deuterium incorporation plots for selected significant peptides shown in panel H.

## Supplementary Figure 7. MbTEC is autoinhibited by its PH domain.

A

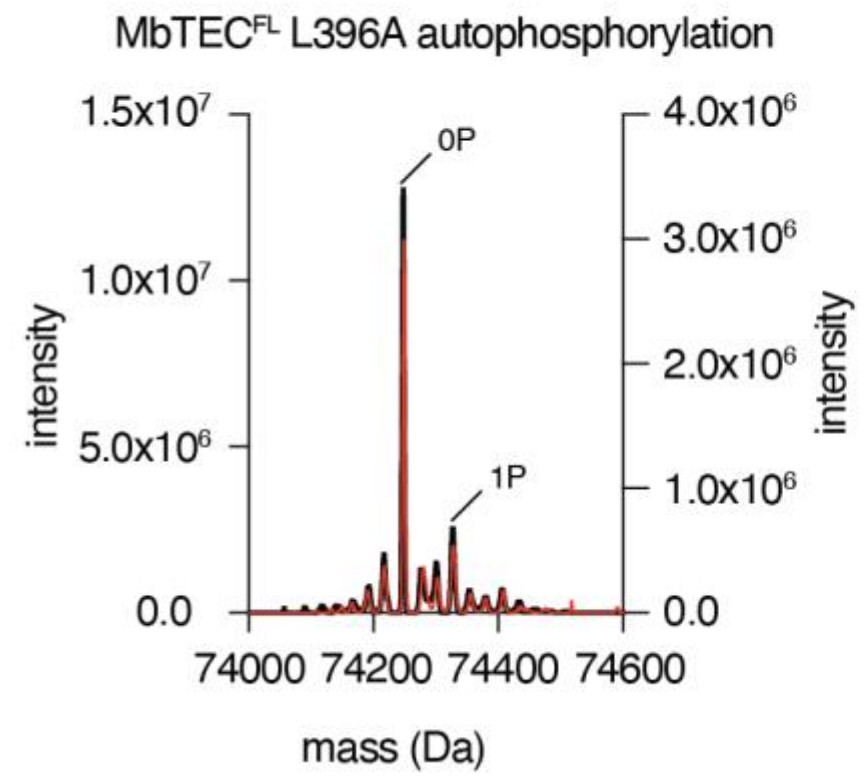


B

MS/MS of autophosphorylated MbTEC L396A (trypsin digest)

MbTEC$^{FL}$ L396A coverage = 85.2%
MbTEC$^{32K}$ L396A coverage = 79.4%

| Position | Sequence | ratio mod/base: MbTEC$^{32K}$ L396A | ratio mod/base: MbTEC$^{FL}$ L396A |
|---|---|---|---|
| 559 | FVIDDEYTASEGTK (2) | 0.03 | 0.00 |
| 559 | FVIDDEYTASEGTKFPIK (2) | 0.30 | 0.02 |
| 559 | FVIDDEYTASEGTKFPIK (3) | 0.42 | 0.04 |

A. Mass spectrometry analysis of the product of MbTEC$^{FL}$ L396A autophosphorylation (denoted by the black circle in panel A). Intensities before autophosphorylation in black (left axis) and after autophosphorylation in red (right axis).

B. Tandem mass spectrometry identification of the modified residues in MbTEC$^{32K}$ L396A.

## Supplementary Figure 8. Membrane translocation of MbTEC$^{FL}$ and MbTEC$^{PH}$ in cells.

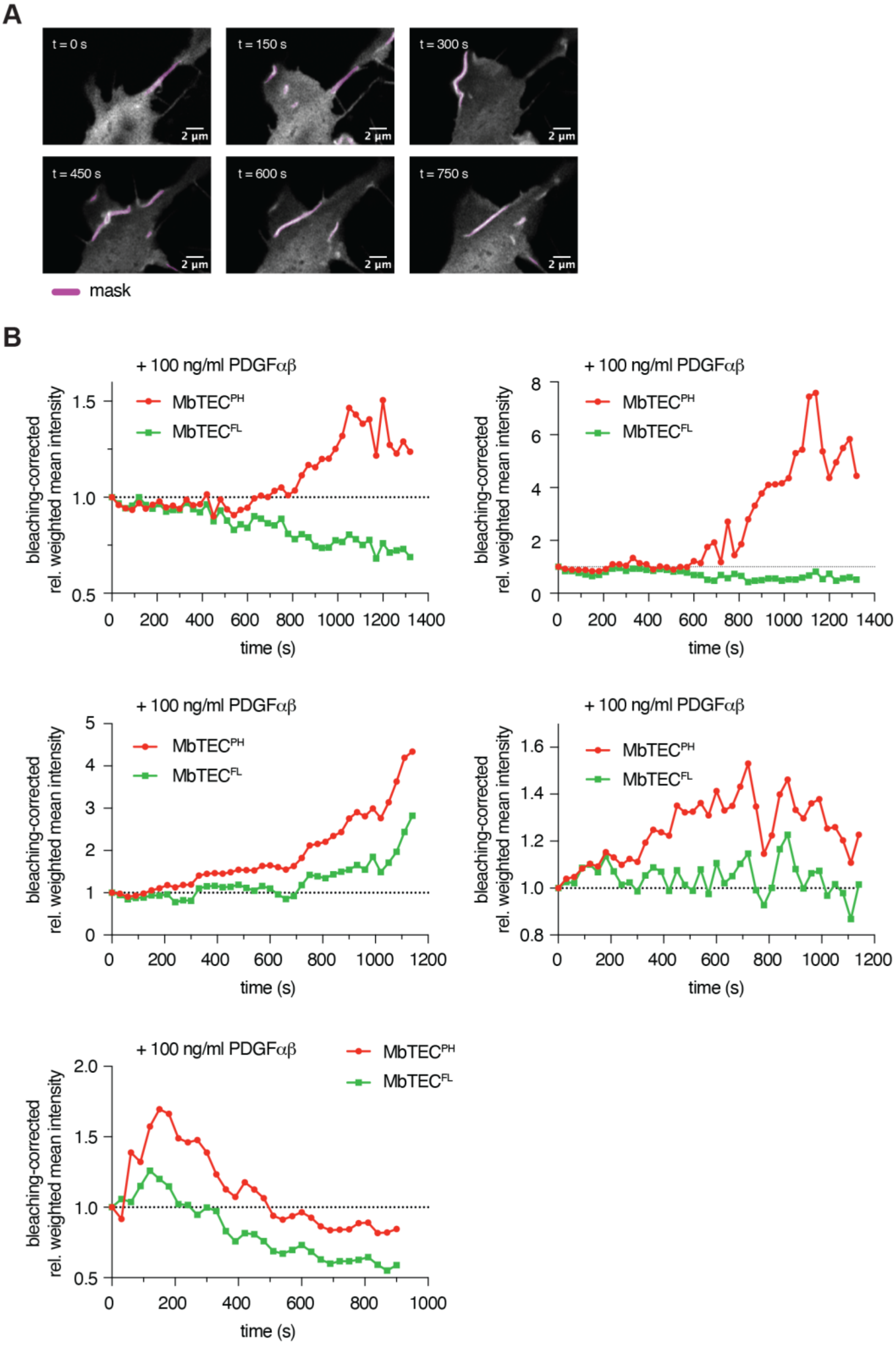


A. Plasma membrane association of EGFP-MbTEC$^{FL}$ and mCherry-MbTEC$^{PH}$ in a representative NIH3T3 fibroblast following stimulation with PDGFαβ. Mask of

filopodia-like membrane structures used for signal integration shown in magenta.

B. Bleaching-corrected, normalized mask intensity profiles of $MbTEC^{FL}$ (green) and $MbTEC^{PH}$ (red) in five independent cells post-PDGFαβ stimulation as a function of time.

## Supplementary Figure 9. MbTEC is allosterically activated by $PIP_3$.

**A**

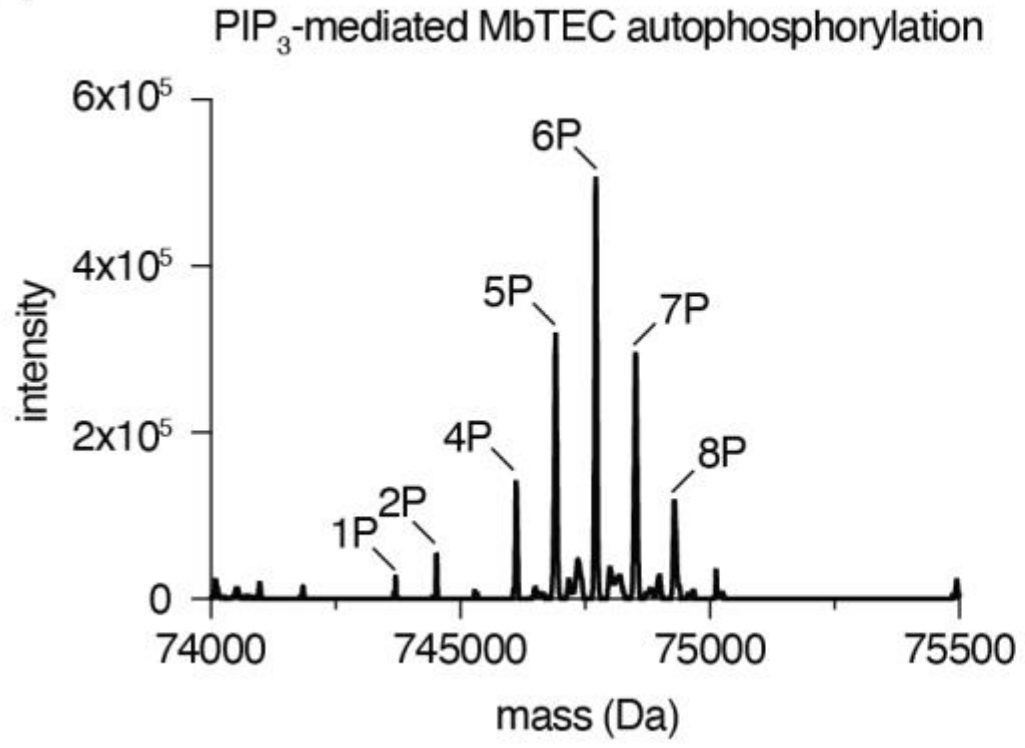


**B**

*MbTEC*

| Position | Sequence window | Probability | Modification multiplicity | # sequences | # modified sequences | Spectral count |
|---|---|---|---|---|---|---|
| 279 | AGQEG**Y**IPANY | 1.00 | 1;2 | 5 | 9 | 157 |
| 284 | YIPAN**Y**VRKLG | 1.00 | 1;2 | 5 | 9 | 131 |
| 336 | SLSVF**Y**QGKLK | 1.00 | 1;2 | 3 | 6 | 10 |
| 352 | HDQDM**Y**RVSER | 1.00 | 1 | 4 | 6 | 22 |
| 369 | SELIE**Y**HKHNG | 1.00 | 1 | 2 | 2 | 11 |
| 427 | VRAGV**Y**KSNRP | 1.00 | 1 | 2 | 2 | 16 |
| 559 | VIDDE**Y**TASEG | 1.00 | 1 | 2 | 2 | 15 |

**C**

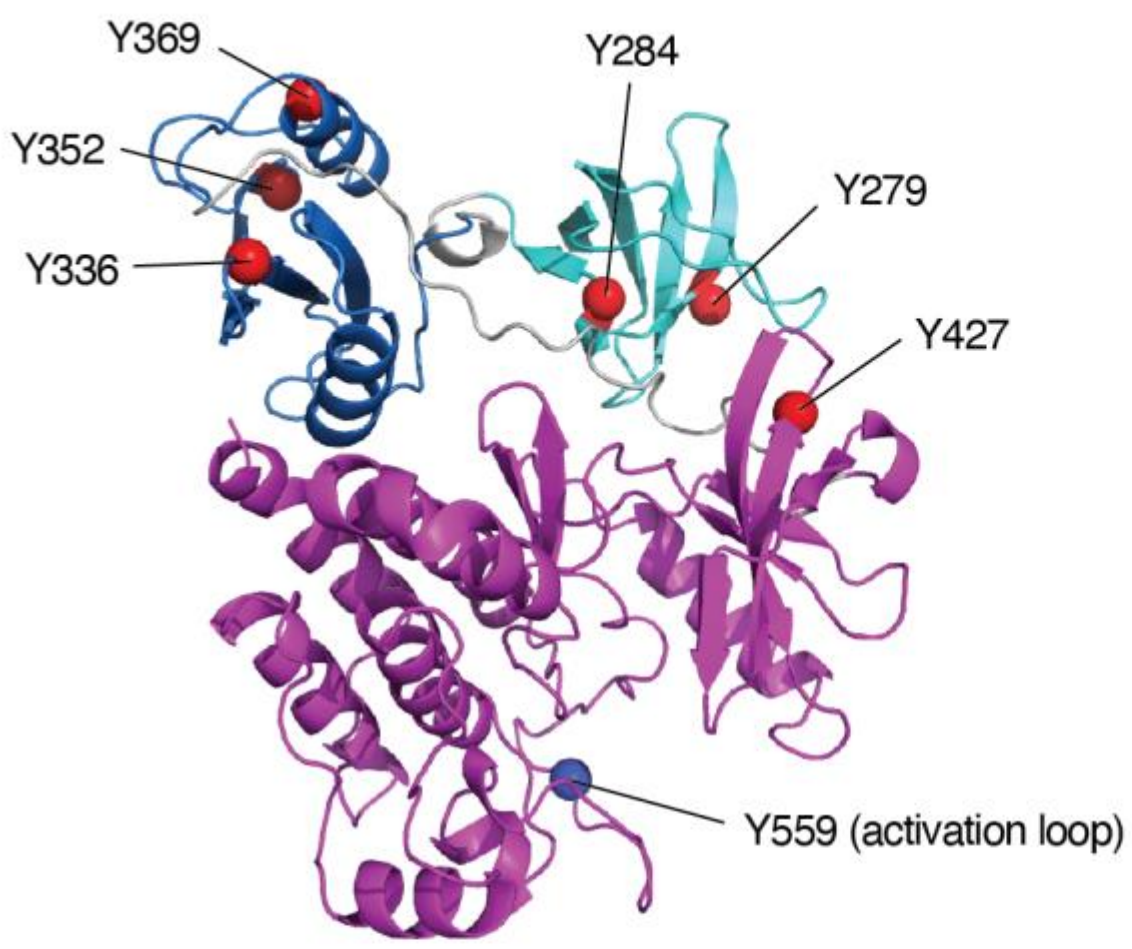


**D**

*Membrane scaffold protein 1 (MSP1D1)*

| Position | Sequence window | Probability | Modification multiplicity | # sequences | # modified sequences | Spectral count |
|---|---|---|---|---|---|---|
| 47 | AKVQP**Y**LDDFQ | 0.99 | 1 | 1 | 1 | 2 |
| 62 | EEMEL**Y**RQKVE | 1.00 | 1 | 5 | 5 | 7 |
| 113 | THLAP**Y**SDELR | 1.00 | 1 | 4 | 4 | 9 |
| 139 | ARLAE**Y**HAKAT | 1.00 | 1 | 3 | 3 | 8 |
| 183 | SALEE**Y**TKKLN | 1.00 | 1 | 2 | 2 | 2 |

A. Intact mass spectrometry of MbTEC after 90 min autophosphorylation on $PIP_3$-containing nanodiscs.

B. Tandem mass spectrometry analysis of autophosphorylated MbTEC following activation by $PIP_3$ nanodiscs.

C. Phospho-sites identified in panel A mapped onto predicted structure of $MbTEC^{32K}$.

D. Phospho-sites in MSP1D1 identified by tandem mass spectrometry.

Supplementary Figure 10. Paralog-specific differences in the PH domain of Tec kinases.

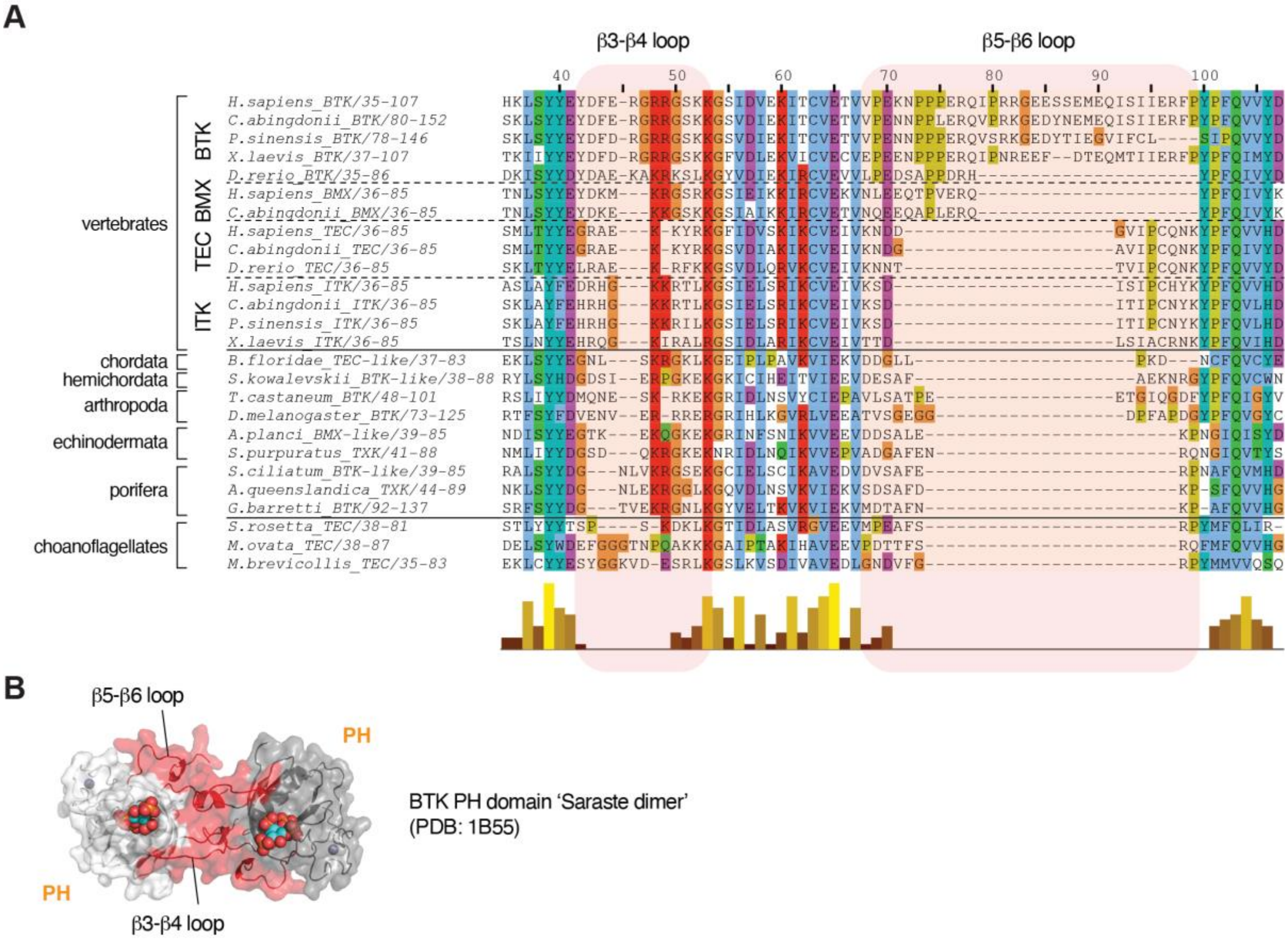


A. Partial multiple sequence alignment of the PH domain of the TEC kinases over 600-800 My of evolution. Loop insertions β3-β4 and β5-β6 specific to BTK are highlighted with a red background.

B. Crystal structure of the 'Saraste' dimer of the BTK PH domain, indicating that the β3-β4 and β5-β6 insertion loops in BTK mediate dimerization.
